# Residues of TRPM8 at the Lipid-Water-Interface have Coevolved with Cholesterol Interaction and are Relevant for Diverse Health Disorders

**DOI:** 10.1007/s00232-024-00319-y

**Published:** 2024-08-16

**Authors:** Deep Shikha, Ritesh Dalai, Shamit Kumar, Chandan Goswami

**Affiliations:** School of Biological Sciences, National Institute of Science Education and Research, An OCC of Homi Bhabha National Institute, Khordha, Jatni, Odisha 752050 India

**Keywords:** Lipid-Water-Interface, CARC-CRAC motifs, TRPM8-Cholesterol interaction, Co-Evolution, Amino acid conservation

## Abstract

TRPM8 is a non-selective cation channel that is expressed in several tissues and cells and also has a unique property to be activated by low-temperature. In this work, we have analyzed the conservation of amino acids that are present in the lipid-water-interface (LWI) region of TRPM8, the region which experiences a microenvironment near the membrane surface. We demonstrate that the amino acids present in the LWI region are more conserved than the transmembrane or even full-length TRPM8, suggesting strong selection pressure in these residues. TRPM8 also has several conserved cholesterol-binding motifs where cholesterol can bind in different modes and energies. We suggest that mutations and/or physiological conditions can potentially alter these TRPM8-cholesterol complexes and can lead to physiological disorders or even apparently irreversible diseases such as cancer and neurodegeneration.

**Graphical abstract:**

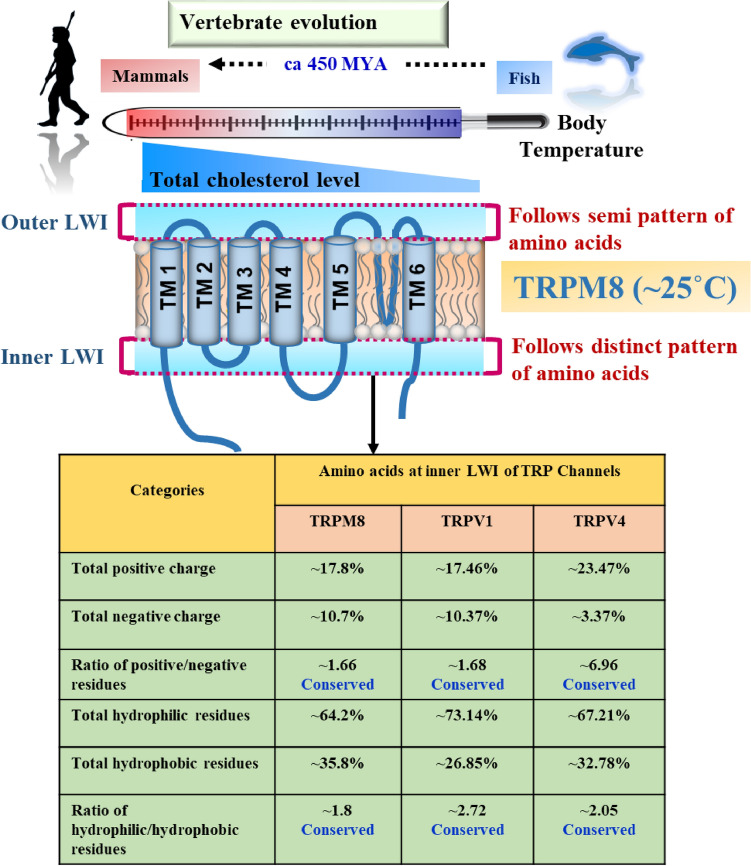

**Supplementary Information:**

The online version contains supplementary material available at 10.1007/s00232-024-00319-y.

## Introduction

Transient receptor potential cation channel subfamily M (melastatin) member 8 (TRPM8) is a non-selective cation channel (Buijs and McNaughton 2020). TRPM8 also has a unique property of thermogating, i.e., it gets activated at low temperatures (< 23 °C) and thus acts as a cold-activated ion channel. TRPM8 is a six-transmembrane channel and is poly-model in nature. TRPM8 can be activated by cooling agents such as menthol, and icilin or at lower temperatures, i.e. at 23–28 °C (Yin et al. 2018; Bautista et al. 2007; Peier et al. 2002). TRPM8 is expressed in peripheral neurons, involved in cold-sensation, and is also involved in several pain-related behaviors (Colburn et al. 2007). However, later studies have indicated that the expression of TRPM8 is not limited only to neurons. It is widely expressed in diverse tissues and cells that are non-neuronal in origin. For example, expression of TRPM8 is observed in T cells, Osteoblasts, Bone Marrow-Derived Mesenchymal Stem Cell pools, Murine Macrophages, etc. (Acharya et al. 2021a, 2021b; Khalil et al. 2016). Therefore, the functional regulation of TRPM8 is very critical for overall physiological conditions. Indeed, the expression of TRPM8 in neuronal and non-neuronal cells is relevant for cold hypersensitivity, nerve injury, chronic pain, and also in prostate cancer (Iftinca and Altier 2020).

During vertebrate evolution, both changes in body temperature (and related physiological changes) and preferred environmental niches played an important role. As TRPM8 is involved in the detection of cold stimulus, the function of TRPM8 is also critical for the evolution of species. This is mainly due to the fact that TRPM8 is involved in precise sensory functions which are useful for avoidance of noxious stimulus and thus will provide adaptive benefits. Therefore, mutations that do not provide adaptive benefits are likely to be non-selected. Thus, critical sequence analysis can indicate the molecular evolution, and selection pressure on specific regions of TRPM8. This analysis can also indicate the mismatches that can induce diseases and/or pathophysiological conditions. Accordingly, experiments with knockout animals show that *Trpm8*^*−/−*^ animals have reduced sensory function in response to low temperature (28 °C), but these animals are not completely insensitive to temperature either (Bautista et al. 2007). *Trpm8*^*−/−*^ animal has been reported to be defective in response to heat sensation (Montesinos et al. 2020). Using wild-type and knockout animals from several labs suggests that the physiological response mediated by TRPM8 are complex and can be regulated by different factors.

So far, a large number of ion channels and receptors are known where membrane cholesterol seems to play role in critical functional regulation (Zakany et al. 2020). At the molecular level, cholesterol also interacts with several membrane proteins and such membrane proteins have putative cholesterol-binding motifs, termed as CARC, CRAC, and CCM-motifs (Fantini and Barrantes 2013). Cholesterol-mediated regulation is critical for several TRP members, and such regulation is relevant for thermogating behavior as well. For example, TRPV1 interacts with cholesterol, and there are certain amino acids present in the lipid-water-interface that are critical for such interaction (Saha et al. 2022, 2017). Level of membrane cholesterol also regulates the temperature thresholds required for TRPV1 activation. Cholesterol reduction reduces the optimum activation temperature whereas cholesterol saturation increases the optimum activation temperature (Liu et al. 2003, 2006). TRPV4 also interacts with cholesterol and other interacting proteins through some conserved motifs and mutations in such motifs correlate well with genetic disorders (Das and Goswami 2019; Das et al. 2022, 2023; Kumari et al. 2015).

Lipid-water-interface (LWI) regions of lipid bilayer are very special microenvironments where the availability of free water is low (but not nil) and is dynamically changing with different conditions. LWI regions also reside many active compounds, and different gases, as several ion channel-specific ligands are preferably phase-separated to some extent and thus become enriched there. Thus understanding of the molecular evolution of LWI regions, factors exerting selection pressure on these regions and the extent of selection provides useful information about the function of ion channels. In this work, we explore the molecular evolution of amino acids located in the lipid-water-interface region of the TRPM8 as well as in different cholesterol-binding motif sequences. We demonstrate that LWI-regions of TRPM8 could be the critical regions in the whole protein structure that might have contributed for TRPM8 selection throughout the vertebrate evolution. Our data indicates that cholesterol could be relevant for the proper function of TRPM8, and the molecular evolution of TRPM8 may have been influenced by cholesterol interaction, preferably at the lipid-water interface regions.

## Material and Methods

### TRPM8 Protein Sequences

All TRPM8 sequences were retrieved from public databases such as [NCBI, https://www.ncbi.nlm.nih.gov/protein/] available on the public platform. The individual accession numbers used for the analysis are also provided in the supplementary table (Supplementary Table 1). The human TRPM8 protein sequence was accessed from Uniprot (accession no: Q7Z2W7). The human sequence was represented using the Protter tool. The N- and C-terminal regions of each transmembrane domain are highlighted in red and green colors, respectively. A total of 67 different TRPM8 sequences from different vertebrate species were retrieved and all were saved in FASTA format (Supplementary Table 1). The sequence logo of amino acid conservation was plotted using the WebLogo online application. The conservation analysis has been performed by aligning the sequences using the MEGA5.1 alignment tool. Boxplot analysis was done by calculating the distance between two amino acids of aligned sequences by using the Bootstrap method as described before (Saha et al. 2017).

### Frequency Plot of TRPM8

A stretch of five amino acids (in both N- and C-terminal direction) from the trans-membrane region of each helix was considered as Lipid Water Interface region (LWI) as mentioned before (Saha et al. 2022; White and Wimley 1998). The outer, inner, and total LWI amino acid percentages were calculated for each amino acid throughout the vertebrate evolution. The frequency of total hydrophobic residues (Trp, Phe, Tyr, Leu, Ile, Cys, Met), total hydrophilic (Ala, Arg, Asn, Asp, Gln, His, Pro, Ser, Thr, Lys, Gly, Val) residues were calculated as described before. Total positive (Arg, Lys, and His) and total negative (Asp and Glu) residues were also calculated, as mentioned previously (Saha et al. 2022). The ratio of positive: negative and hydrophobic: hydrophilic was plotted in GraphPad Prism 7. Mann–Whitney test was performed to calculate statistical significance.

### Conservation Analysis of Cholesterol-Binding Motifs in TRPM8

The cholesterol-binding motif CRAC (cholesterol recognition amino acid consensus) and CARC (invert sequence of CRAC) were searched in the full-length TRPM8 protein structure by using their cholesterol consensus motifs (CCM). The consensus motif for CRAC, CARC and CCM are [L/V]-(X)(1–5)-Y-(X)(1–5)-[R/K], [R/K]-[X](1–5)-[Y]-[X](1–5)-[L/V] and [R/K]-X(2–6)-[I/V/L]-X3-(W/Y) respectively (Fantini and Barrantes 2013; Saha et al. 2017). All the sequences representing CARC or CRAC motifs present in the TRPM8 are listed (Supplementary Table 2).

### *Cancer* mutation analysis in TRPM8

Online web page (https://cancer.sanger.ac.uk/cosmic) COSMIC that stands for the Catalogue for Somatic Mutations in Cancer, was used to obtain all the somatic cancer mutations that are present in TRPM8 protein till February, 2022. All the mutations that have been reported so far in the CARC-CRAC regions and LWI regions of full-length TRPM8 protein were obtained (Supplementary Table 3) and marked in the “open” and “closed” conformation of TRPM8 Cryo EM structures (6NR2 and 6O6R respectively).

### Cholesterol Docking with TRPM8

The avian (6NR4, 6NR2, 6NR3, 6BPQ, 6O6A, 6O77, 6O7R, 6O72), mouse (7WRA, 7WRB, 7WRC, 7WRD, 7WRE, 7WRF, 8E4L, 8E4M, 8E4N, 8E4O, 8E4P) and human (8BDC) TRPM8 structures were obtained from PDB and a PDBQT file of receptor has been created (Yin et al. 2019, 2022; Diver et al. 2020; Yin et al. 2019; Zhao et al. 2022; Palchevskyi et al. 2023). The structure of ligand i.e., Cholesterol, has been downloaded from the PubChem website (https://pubchem.ncbi.nlm.nih.gov/compound/5997). PDB file of cholesterol has been created from the SDF format using Open Babel GUI. Docking grid data has been obtained by opening both ligand and receptors in Autodock 4. The docking has been performed with Autodock Vina at an exhaustiveness of 32 for better refinement (Eberhardt et al. 2021).

### Boxplot Analysis

Boxplot representation was used to show the conservation/divergence of transmembrane domains of TRPM8 as well as for the different cholesterol-binding motifs (CARC and CRAC) sequences. The Y-axis represents the conservation/divergence of the domain. The lesser the value in Y-axis, the higher the conservation of regions, as described before (Sardar et al. 2012). The highly conserved protein Histone-4 was plotted alongside in the box-plot which sequence was retrieved from the ENSEMBL database. The median values and significance of the data were tested by the Kruskal–Wallis test in "R" software for boxplot. The GraphPad Prism 7 was used to plot the graphs.

### Chemicals and Reagents

WS12 (Tocris) was used as a TRPM8 activator, and AMTB (Tocris) as an inhibitor. DMSO (Sigma) was used as a vehicle control. For staining purposes anti TRPM8 antibody (Alomone Labs).

was used. For lipid raft staining purposes, Alexa Fluor-594 conjugated Cholera Toxin-B (Invitrogen) was used. The nucleus was stained with DAPI (Invitrogen). The anti-rabbit secondary antibody AF-488 was procured from Invitrogen.

### Cell Culture and Immunostaining

Peritoneal macrophage was isolated by following as per the IAEC approval (NISER/SBS/IAEC/AH-229). 4–6 weeks-old mice were euthanized, and macrophage cells were isolated from the peritoneal cavity in chilled 1XPBS (Phosphate buffer saline), as per the protocol (Mahish et al. 2023). Further, cells were seeded on 25 mm coverslips (VWR) and supplemented with complete RPMI media (Invitrogen) supplemented with 10% Fetal Bovine Serum (Gibco) antibacterial-antifungal agents Penicillin–Streptomycin (HiMedia) and Amphotericin- B (Sigma). Cells were grown at 37 °C with 5% CO_2_ in an incubator. Three biological repeats of the experiments have been performed. For experiment purpose, TRPM8 modulators were added for total 4 h (3:30 h alone and last 30 min with or without β-MCD where applicable). After incubation, cells were fixed with 4% PFA. For immunostaining, cells were washed thrice with 1XPBS and left unpermeabilized to study the surface expression only. Cell were further stained with TRPM8 antibody (1:500 dilution) overnight. Next day, the cells were washed with 1XPBS and incubated with anti-rabbit antibody AF-488 (1:1000) and with CTxB-594 (1:500) for 2 h. At last, the nucleus was stained in 1:1000 concentration for 15 min. Cells were mounted on the coverslip with the mounting agent Fluoromount-G (SouthernBiotech).

### Imaging, Quantification, and Analysis

All images were acquired by Olympus, FB3000 confocal microscope with 60X objective. Argon laser was used for TRPM8 antibody (Ex/Em: 488/560 nm) and CTxB-594 (Ex/Em: 590-618 nm) at the same time to acquire both the intensity for a single cell. For quantification purposes, 15 cells from each biological repeat (45 cells from 3 sets) were quantified in FIJI software. The region of interest was drawn manually around the cell and the area as well as intensity of TRPM8 as well as CTxB have been quantified for individual cells. For each set, all the conditions have been normalized with the median value of the vehicle control (DMSO). The three sets of normalized values have been plotted in GraphPad Prism 9. For statistical significance, a non-parametric Kruskal–Wallis test was performed.

## Results

### TRPM8 has Highly Conserved Amino Acid Sequences in Lipid-Water-Interface Regions

**Fig. 1:**
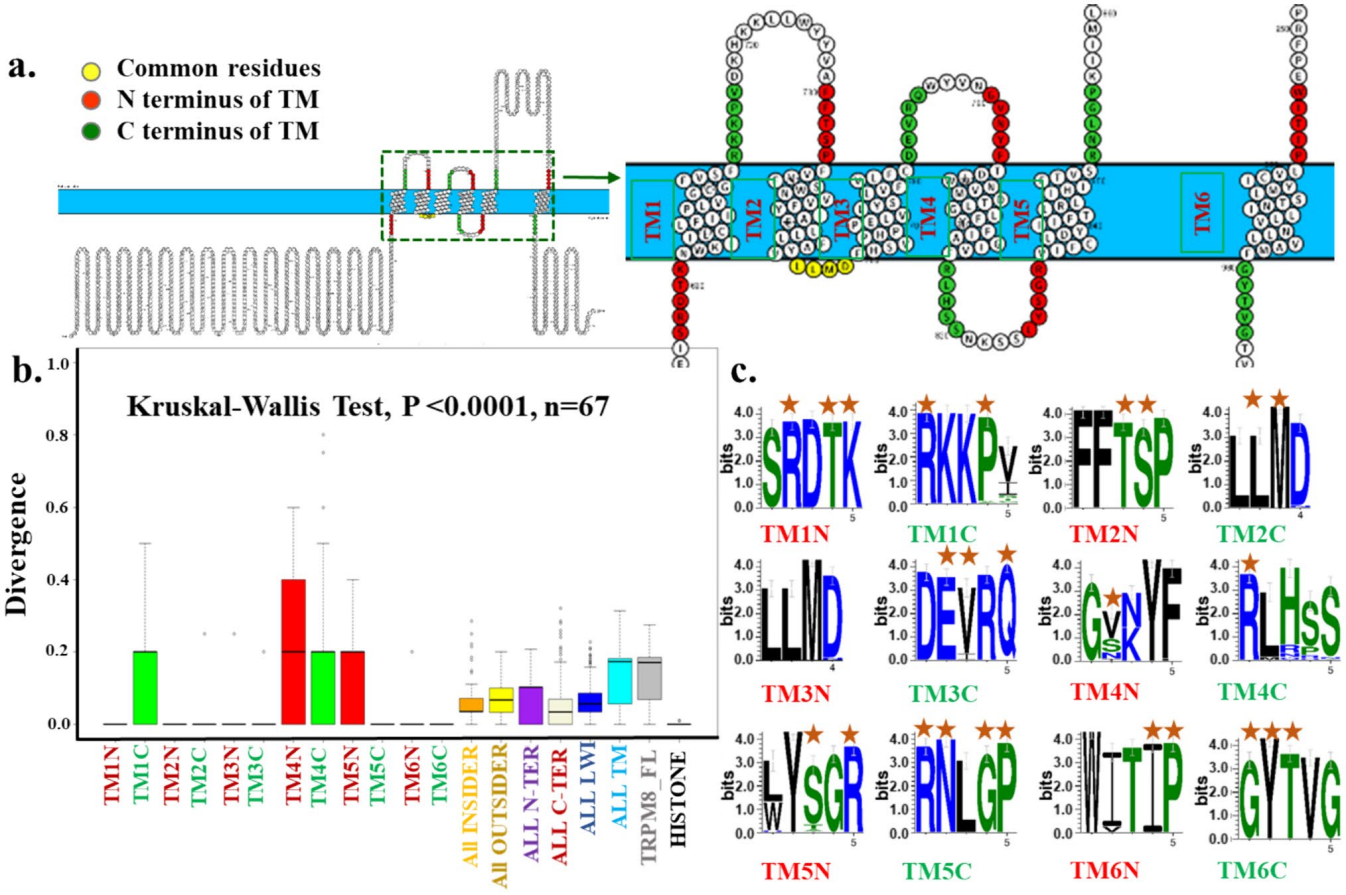
Amino acids present in the lipid-water-interface (LWI) regions of TRPM8 are highly conserved. **a.** Schematic representation of full-length human TRPM8 protein. The zoomed figure on the right side depicts the transmembrane regions of TRPM8, where green and red color represent N- and C-terminus LWI regions for each transmembrane sequence, respectively. The LWI regions located between TM2 and TM3 have overlapped with each other to some extent and are thus marked with yellow color. **b.** Conservation analysis of all the LWI regions separately, all inside, all outside, all N-terminal, and all C-terminal regions are shown. All LWI regions together remain more conserved than the full-length or even all TM regions. Conservation of Histone H4 has been considered as a positive control. **c.** Amino acids of different LWI regions are highly conserved throughout vertebrate evolution as shown by seqlogo analysis. Many of the conserved amino acids are also the hotspots for cancer-related mutations (shown by red star marks)

To study the importance of the LWI regions in TRPM8 evolution, we performed a conservation analysis using all vertebrate sequences. We have selected a stretch of five amino acids at the N- and C-terminal of each TM region which is approximately 6–10 Å in length (Fig. 1a). A total of 8 out of 12 LWI regions remain highly conserved, suggesting the importance of the LWI region in the overall channel function (Fig. 1b). Notably, the inner LWI regions are more conserved than the outer LWI region (stat value, *p* =  < 0.0001, unpaired non-parametric Mann–Whitney t-test). This indicates the overall selection pressure on the inner LWI region was more than the outer LWI region. This may also suggest that the factors that are present in the cytosolic region and the lipids that are present in the inner leaflet of the membrane have exerted more selection pressure on TRPM8 function. We also noted that overall, the LWI regions are more conserved than the TM regions or even full-length TRPM8 (stat value, *p* =  < 0.0001, unpaired non-parametric Mann–Whitney t-test), suggesting that LWI regions have more selection pressure than the TM or even full-length and are functionally more critical than the TM regions (Fig. 1b). The LWI representing the C-terminal peptide sequence is more conserved than the N-terminal sequence (stat value, *p* =  < 0.0001, unpaired non-parametric Mann–Whitney t-test) suggesting that the peptide directionality also plays a role in this selection.

Next, we analyzed the seqlogo of all the LWI regions and compared that with mutations in TRPM8 that are listed in cancer patients which include lymphoid neoplasm, carcinoma, malignant melanoma, pancreatic intraepithelial neoplasia, hematopoietic neoplasm, carcinoid endocrine tumor, primitive neuroectodermal tumor- medulloblastoma, glioma etc. (Fig. 1c). We noted that several amino acids that are present in the LWI regions and are highly conserved have mutated in the case of cancer patients. Analysis of these mutations also suggests that the frequency of cancer is also observed to be higher in the LWI region. The frequency of the cancer mutation observed in the LWI region is ~ 61% (for 60 amino acids) and the frequency of the cancer mutation observed in the rest of the TRPM8 regions is 39% (1104–60 = 1044 amino acids). Which suggests that the LWI can actually serve as a potential “hotspot for cancer”. This strongly suggests the importance of TRPM8 in cancer and also suggests somatic mutations in TRPM8 at the LWI region as a possible causal factor for cancer progression (discussed later).

### Analysis of Amino Acid Frequencies at the Lipid-Water-Interface Region of TRPM8

**Fig. 2:**
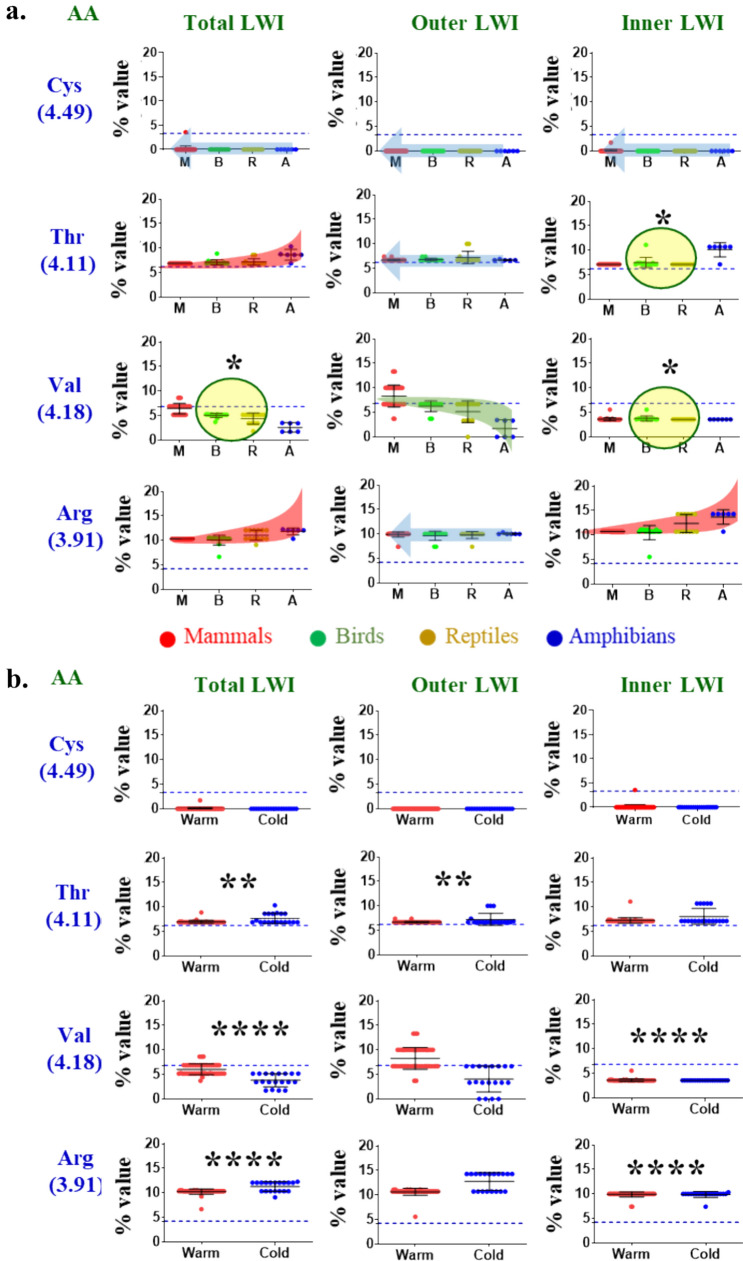
Frequency analysis of residues at the LWI region indicates the trends of conservation, exclusion, positive and negative-selection during vertebrate evolution. **a**. Frequency analysis of Cys, Thr, Val, Arg are shown here (for all 20 amino acids, see supplementary Fig S1). Frequencies that remain conserved are shown by blue arrow, positive selection by a green background and negative selection with a red background. Certain residues show zero frequency throughout the vertebrate evolution suggesting complete exclusion of such amino acids (such as Cys) from the LWI region. The dotted line indicates the expected natural frequency of that amino acids. The green circle indicates the sudden increase in frequency from reptiles to birds (i.e. possible involvement of these residues for adjusting the channel function in altered body temperature). * = *p* < 0.01. **b.** Graphical representation of frequencies of amino acids in warm (red dots) and cold-blooded (blue dots) animals are shown. The dotted-line indicates the expected natural frequency of that amino acids. Mann–Whitney test was performed to calculate statistical significance. The values are: **** = *p* < 0.0001, ** = *p* < 0.001

To gain the details of the molecular evolution of TRPM8 residues, we tested the frequency of all 20 amino acids in the LWI regions in different phylogenetic groups. We could not find any fish-specific TRPM8 sequence from the available databases. This limits us to perform our analysis in amphibian (A), Reptilian (R), Birds (B) and mammals (M) sub-groups. We noted that TRPM8 is missing in most of the fishes (Kastenhuber et al. 2012; Saito and Tominaga 2015; York and Zakon 2022). Systematic analysis of the residues present in all the LWI regions are summarized (Fig. 2, for details, see Supplementary Fig S1).

***Complete exclusion:*** We observed that several amino acids are completely excluded in the LWI region of TRPM8 sequences, suggesting that during the vertebrate evolution, these sequences never appeared in the LWI regions. For example, His, Ala, Met, Cys residues are excluded in the outer LWI region. Comparably, Glu, Gln, Pro, Asn, Ala, Cys, Ile, Phe, residues are excluded from the inner LWI region. Notably, Ala and Cys residues are totally excluded from both outer as well as inner LWI regions.

***Positive selection:*** We noted that certain amino acids become positively selected, i.e. the trend of their frequency increased gradually during the vertebrate evolution. For example, Val in the outer LWI region has increased from lower vertebrates to mammals. In the same notion, His in the inner LWI region has increased from lower to higher vertebrates. Considering both outer and inner LWI regions, a positive selection of Val in total LWI is observed.

***Negative selection:*** Opposite to positive selection, we observed that certain amino acids in specific regions have decreased during vertebrate evolution. For example, Ile has have declined trend infrequency in the outer LWI region. At the inner LWI region, Arg has declined trend in frequency during the vertebrate evolution. Considering both inner and outer LWI regions, Arg, Thr, and Ile residues have declined trend in frequency during the vertebrate evolution.

***Amino acids with conserved frequencies:*** We noted that several amino acids are neither excluded nor selected positively or negatively. These amino acids remain at a conserved frequency during the vertebrate evolution. In other words, the presence of these amino acids in these frequencies is optimum, and critical for the channel function. We noted that at the outer LWI, there are amino acids such as Glu, Asp, Pro, Arg, Thr, Gly, Leu, Tyr, Phe, and Trp that remain at conserved frequencies. In a similar manner, at the inner LWI regions, a few amino acids, such as Lys, Val, Gly, and Tyr, remain at conserved frequencies. At the inner LWI region, Asp residue also remains at a conserved value though with some out layers. Considering both inner and outer LWI regions, Glu, Asp, Gln, Pro, His, Gly, Met, Tyr, and Phe remain mostly conserved during vertebrate evolution. Notably, in the case of TRPM8, overall, more amino acids are conserved at the outer LWI than the inner LWI region (discussed later).

***Amino acids that remain more than the natural frequencies:*** While certain amino acids remain at conserved frequencies, there are cases when these residues appear more than the natural frequencies, suggesting more selection pressure at these amino acids. For example, at the outer LWI region, Pro, Arg, Phe, and Trp remain at higher values than the natural frequencies. Similarly, at the inner LWI region, Asp, Thr, Gly, Met, Leu and Tyr remain at higher values than their respective natural frequency. Arg also appears more than its natural frequency (with a trend of negative selection). Considering both inner LWI and outer LWI regions, Asp, Pro, Thr (With a trend of negative selection), Gly, Met, Tyr, and Phe appear more than their respective natural frequencies.

***Amino acids that remain less than the natural frequencies:*** We noted that a few amino acids remain conserved throughout the vertebrate evolution, yet maintained at lower than the natural frequencies. This includes Glu, Asp, and Leu in the outer LWI regions. Similarly, Lys and Val remain at the less then natural frequencies at the inner LWI region. Considering both inner and outer LWI regions, Glu, Gln, and His are retained at lower than natural frequency.

***Amino acids that remain just equal to the natural frequencies:*** We noted that few amino acids remain conserved throughout the vertebrate evolution and their frequency match just equal to the natural frequency. These include Thr, Gly, and Tyr at the outer LWI region only.

All these analyses provide important information about the possible regulation of TRPM8 by Cys modification, glycosylation, and phosphorylation (discussed later).

### Comparatives of LWI Residues in Human TRPM8 as Derived From Protter vs Cryo EM

Recently the Cryo-EM structure of human TRPM8 (PDB ID: 8BDC) become available (Palchevskyi et al. 2023). This allowed us to compare the TM and LWI residues as derived from Protter and in the Cryo-EM structure. We found that majority of the TM and LWI residues detected by these two different approaches match well (Supplementary Fig S2).

### Ratio of Positive–Negative Residues and Hydrophilic-Hydrophobic Residues are Conserved in the Inner Lipid-Water-Interface Region of TRPM8

**Fig. 3:**
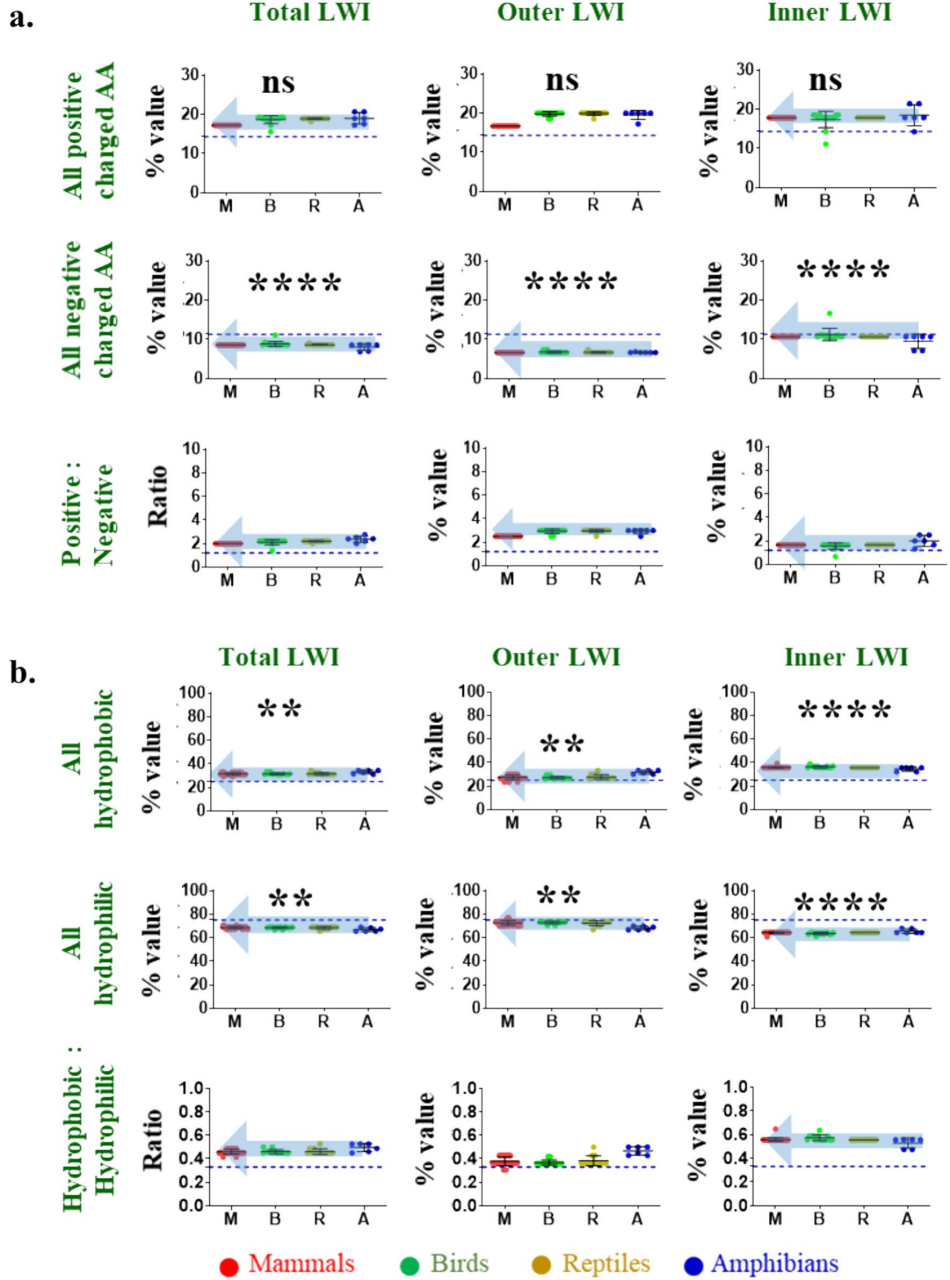
The LWI region of TRPM8 has a unique pattern of amino acids. Shown are the ratio of frequency of hydrophobic, hydrophilic positive and negative amino acids remained conserved during vertebrate evolution. **a.** The graphs represent the total frequency of all positive, all negative and their ratio values in each species throughout the vertebrate evolution. The ratio remains conserved in the inner LWI region and semi-conserved in the outer LWI region. **b.** The frequency of total hydrophobic, total hydrophilic amino acids as well as they remain conserved throughout the vertebrate evolution. This conservation in ratio is observed in the inner LWI region, but not in the outer LWI region. Mann–Whitney test was performed to calculate statistical significance. The values are: **** = *p* < 0.0001, ** = *p* < 0.001

We calculated the frequency of total positively charged and total negatively charged residues, in the inner LWI region. In the inner LWI region, these values remain conserved (total positive charged residue =  ~ 17.8%, negatively charged residue =  ~ 10.7%) throughout the vertebrate evolution (Fig. 3a). We measured the ratio of positive to negative charged residues. Notably, the ratio remains conserved (positive to negative ratio at the inner LWI region =  ~ 1.6) in most vertebrate sequences tested. Similarly, we calculated the frequency of total hydrophobic (Trp, Phe, Tyr, Leu, Ile, Cys, Met, total value is 35.8%) and total hydrophilic residues (Ala, Arg, Asn, Asp, Gln, His, Pro, Ser, Thr, Lys, Gly, Val, total value is 64.2%) in inner LWI regions (Fig. 3b). This ratio also remains conserved (hydrophobic-hydrophilic at the inner LWI region =  ~ 1.8).

The frequency of all negatively charged residues at outer LWI remains conserved (~ 6.6). However, positively charged residues occur at higher frequencies (> 15) and remain conserved with some variations. Notably, the ratio of positive–negative at outer LWI is not conserved.

However, the total (inner and outer LWI regions together) frequency of hydrophobic residues remains conserved (~ 31). Similarly, the total frequency of the hydrophilic residues remains mostly conserved (~ 68.3). However, the ratio of hydrophilic to hydrophobic residues is less conserved as compared to the inner LWI. Similarly, the ratio of positive–negative charged residues is also not conserved when both inner and outer LWI are considered. These findings suggest that during vertebrate evolution, TRPM8 has a conserved pattern of amino acids, especially in its inner LWI region.

### LWI Regions have Very Few Residues that Show Changes due to Body Temperature

During vertebrate evolution, warm-blooded animals evolved from cold-blooded animals and thus core body temperature increased several degrees. As TRPM8 is a temperature-gated on channel, changes in core body temperature is expected to induce changes in its channel opening properties. Thus, a change in core body temperature should be accompanied by changes in TRPM8 sequence so that it will readjust its function in different body temperature. So we hypothesized if a specific amino acid frequency is changed at the LWI region from cold-blooded animals to warm-blooded animals, or even a sudden change from reptilian to birds, then such changes may be triggered by the change in core body temperature. For that we have grouped all the cold-blooded animals (amphibians and reptiles) and warm-blooded animals (birds and mammals) in two distinct groups. We observed that only very few amino acids have significant and notable changes in terms of body temperature and most of the amino acids do not have any change (Supplementary Fig S3).

In that context, we observed that notable changes in Val in the outer LWI region between cold- blooded animals to warm-blooded animals. At the outer LWI region, Thr and Ile also have certain changes between these two groups. In the same context, Leu has notable changes in the inner LWI region. Asp has certain changes between these two groups. Considering both inner LWI and Outer LWI region, Val has notable changes. Other amino acids such as Asp, Thr, Ile, and Leu has certain changes. Therefore, these changes can be relevant for the altered thermogating behavior of TRPM8. Among all these, sudden change in Asn and Ser are observed between reptilians with birds.

Since the “cold response” of avian TRPM8 is very different from that of mammals, especially by its ability to get activated by low temperatures (Pertusa et al. 2018; Yang et al. 2020; Lu et al. 2022), we have also evaluated the amino acid differences in LWI regions between birds and mammals. We have found percentage values of Leucine and Tryptophan are significantly different in the inner LWI region. However, these two amino acids remained unchanged with respect to mammals in the outer LWI region. The total change also remained significantly different for Leu and Trp. On the other hand, a few amino acids, namely Lys, Asn, and Ser remained unchanged in the inner LWI region but were significantly changed at the outer LWI regions (Fig S1). These subtle differences can be important for the differences in the thermal activation of TRPM8 in these two groups.

### TRPM8 has Several Cholesterol-Binding Conserved Motif Sequences

Cholesterol is a plasma membrane component and it impacts several transmembrane proteins. Cholesterol is also a vertebrate-specific molecule. Several membrane proteins show the presence of cholesterol binding. For that, we have used 67 vertebrate-specific TRPM8 sequences and analyzed the occurrence of possible cholesterol-binding sequences in TRPM8.

**Fig. 4:**
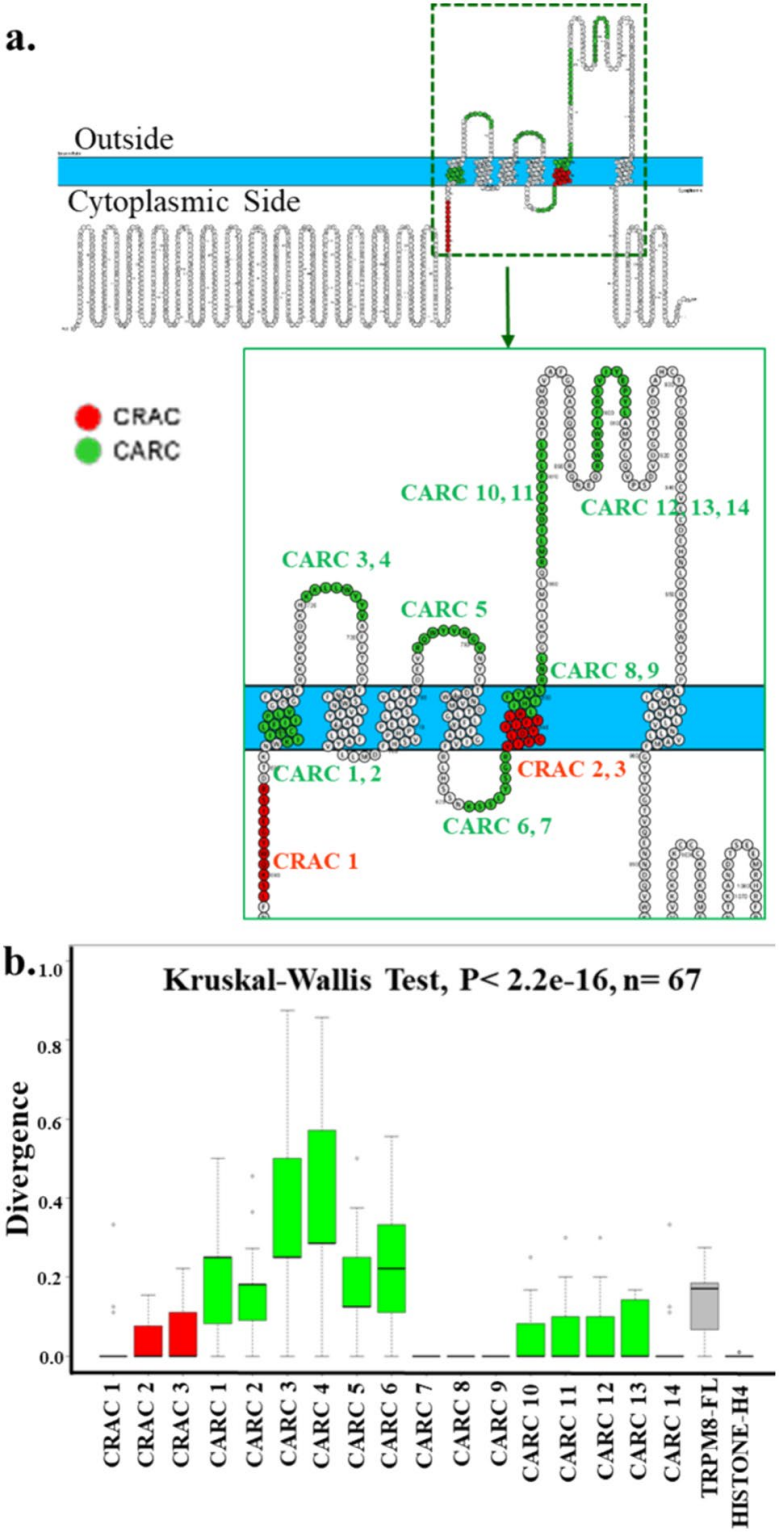
TRPM8 has several cholesterol-binding motif sequences in its LWI and TM regions which are conserved throughout the vertebrate evolution. **a.** Protter image demonstrating the presence of cholesterol recognizing sequences CARC and CRAC in the full-length TRPM8. The zoomed image in the right side shows the CRAC and CARC motifs in red and green respectively. **b.** The box plot shows divergence of different CARC (indicated in red) and CRAC (indicated in green) motifs. Several CARC- and CRAC-motifs present in TRPM8 remain conserved in all vertebrates. Histone H4 sequence has been used a control for highly conserved protein

We observed a total of 40 CARC and 15 CRAC motifs that are present in the human-TRPM8 (Supplementary Table 2). Out of these, 14 CARC and 3 CRAC motifs are located at the TM and LWI regions suggesting that these regions have a high probability of interaction with cholesterol present in the membrane (Fig. 4a). We analyzed the conservation of these potential cholesterol-binding motifs. We observed that 1 CRAC motif (AA 678–688), 4 CARC motifs (7th, 8th, 9th, and 14th motifs, 8th and 9th motifs have overlapping regions) (AA 829–834, 842–853, 842–849, 901–909) are highly conserved (Fig. 4b). Except for the 1st, 3rd, 4th, and 6th CARC motifs, all these motifs have higher conservation than the full-length TRPM8, suggesting that cholesterol has a strong selection pressure on these motifs (discussed later).

### Cholesterol Interaction with TRPM8 Differs with its Different Conformations

To analyze the possible interaction of cholesterol with TRPM8 and further regulation of TRPM8 by cholesterol, we performed docking of cholesterol on avian and human TRPM8. For this, Cryo-EM structure of TRPM8 with or without different ligands were used [PDB: 6BPQ (without any ligand), 6NR2 (with occupancy WS12), 6NR3 (with high occupancy Icilin with PIP_2_ and Ca^2+^), 6NR4 (with low occupancy Icilin with PIP_2_ and Ca^2+^), 6O6A (ligand-free), 6O6R (inhibitor AMTB-bound state), 6O72 (TC1-bound state), 6O77 (Ca^2+^-bound state) and 8BDC (human apo-TRPM8 in a closed-state) (Palchevskyi et al. 2023).

**Fig. 5:**
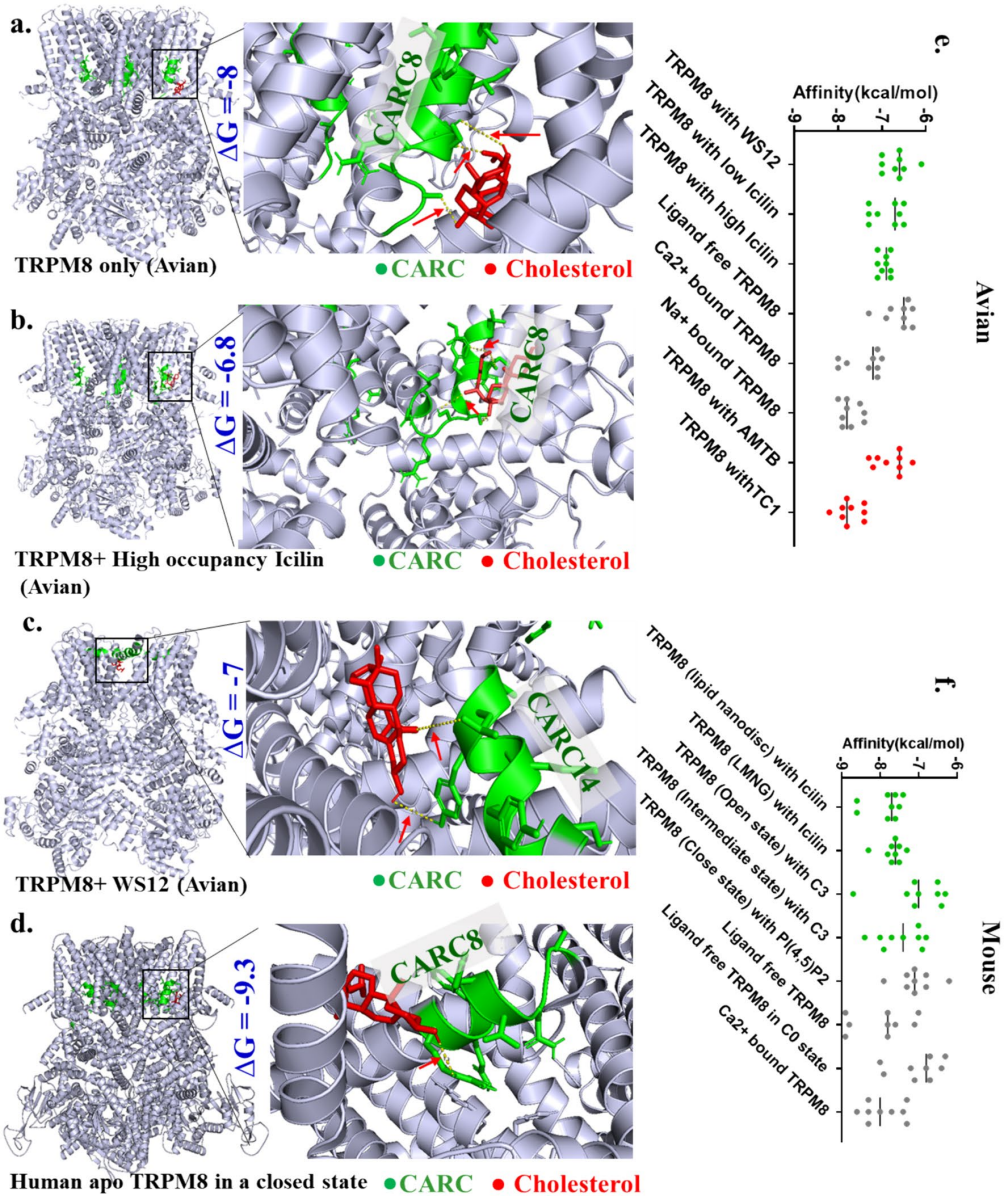
Cholesterol interacts with TRPM8 in apo- and in ligand-bound forms. **a-d.** Shown are the molecular docking of cholesterol (indicated in Red) with the Cryo-EM structures of Avian-TRPM8 (a-c) and Human TRPM8 (d) in different conformations. The respective binding energies (delta G value) are also indicated. **e–f.** Graphical representation of delta G values of cholesterol-binding with TRPM8 in different conformations of bird (d) and mouse (e). For each conformation, top 10 binding modes are plotted here

We observed that cholesterol interacts with avian TRPM8 (without any other ligands, PDB: 6BPQ) and good interaction has been observed at 8th CRAC-motif (one of the highly conserved motifs) (Fig. 5a). Cholesterol interaction is observed on the 8th CRAC-motif of TRPM8 in presence of activator also (with high occupancy Icilin, PDB ID: 6NR3) (Fig. 5b). Cholesterol interaction is also observed on the 14th CARC (another highly conserved motif) in the presence of another activator (WS12, PDB ID: 6NR2) (Fig. 5c). In all these cases H-bond formation between the OH-group of cholesterol with TRPM8 is observed. However, such H-bond formation is not observed in the case of TRPM8 with inhibitor-bound form (with AMTB, PDB ID: 6O6R) (data not shown).

We have performed cholesterol docking with Human TRPM8 (in closed state, 8BDC) as well. Cholesterol docks well at the highly conserved region i.e. CARC8 (Fig. 5d). Cholesterol also docks well on the mouse TRPM8 (ligand-free) at CARC12 with a ∆G value of -8.9 (Fig S5). We have also found interaction of cholesterol at different CARC- and CRAC-motifs in ligand-bound form as well as ligand-free form in avian and mouse TRPM8 (data not shown).

Cholesterol also interacts well with mouse TRPM8 in ligand-free form, at CARC12 (**Fig S4**). In addition to this, cholesterol interaction was also observed with different conformations of TRPM8 in mouse. To analyze the changes in binding energy in different conformations, we calculated the top 10 binding modes of cholesterol with all available conformations of birds (Fig. 5e), and mice (Fig. 5f). Changes in binding energy are observed in the case of different confirmations, suggesting that cholesterol can interfere with the ligand bindings and vice versa.

The cholesterol docking in humans, mice, and avian TRPM8 indicates that CARC8 and CARC14 could be the "hot-spot" for possible cholesterol binding in all these three species. Notably, these 2 regions also remained highly conserved throughout the vertebrate evolution (Fig. 4b). From the data obtained from the Catalogue for Somatic Mutations in Cancer or COSMIC, we have observed that CARC8 and CARC14 regions have various somatic cancer mutations. We have found 4 cancer mutations (i.e., p.R842K, p.R851I, p.N852S, p.N852K) in the CARC8 region, while 6 somatic cancer mutations (i.e., p.R901C, p.S902*, p.S902L, p.I904M, p.Y905 = , p.L909M [Here, * symbol specifies nonsense mutation while ( =) is coding silent] are found in the CARC14 region. Recently interaction of cholesterol on the 3rd extracellular loop has been reported (Lee 2019). Therefore, we compared the cholesterol docking on the 3rd extracellular loop as well, especially on the CARC and CRAC motifs. We observed that in mouse TRPM8, cholesterol forms H-bond (with Glu953) on the 3rd extracellular-loop, especially in the ligand-free conformation (7WRA). We could not observe any possible H-bond formation in any other conformation. In other word, this may also suggest that the loss-of-interaction of cholesterol may prefer channel opening as observed in certain cases of TRPV1 and TRPV4 (Saha et al. 2022; Das and Goswami 2019). In our docking experiments (with Flycatcher TRPM8-cholesterol docking) we only focused on CARC-CRAC motifs, which we found to have several common sequences that matches with the Lee 2019 study (Eberhardt et al. 2021). Some notably residues are Leu842 from CARC-8, Val902 and Leu908 from CARC-14. Taken together the data suggests that CARC8 and CARC14 remain highly conserved and most prominent regions for cholesterol interaction. These two regions might have played a crucial role in TRPM8 evolution. These two regions may also have role in fine-tuning of TRPM8-specific function/s during the vertebrate evolution, and cholesterol interaction may have played a crucial role in such selection.

### Alteration in the Cholesterol Level Affect the TRPM8 Localization on the Cell Surface

**Fig. 6:**
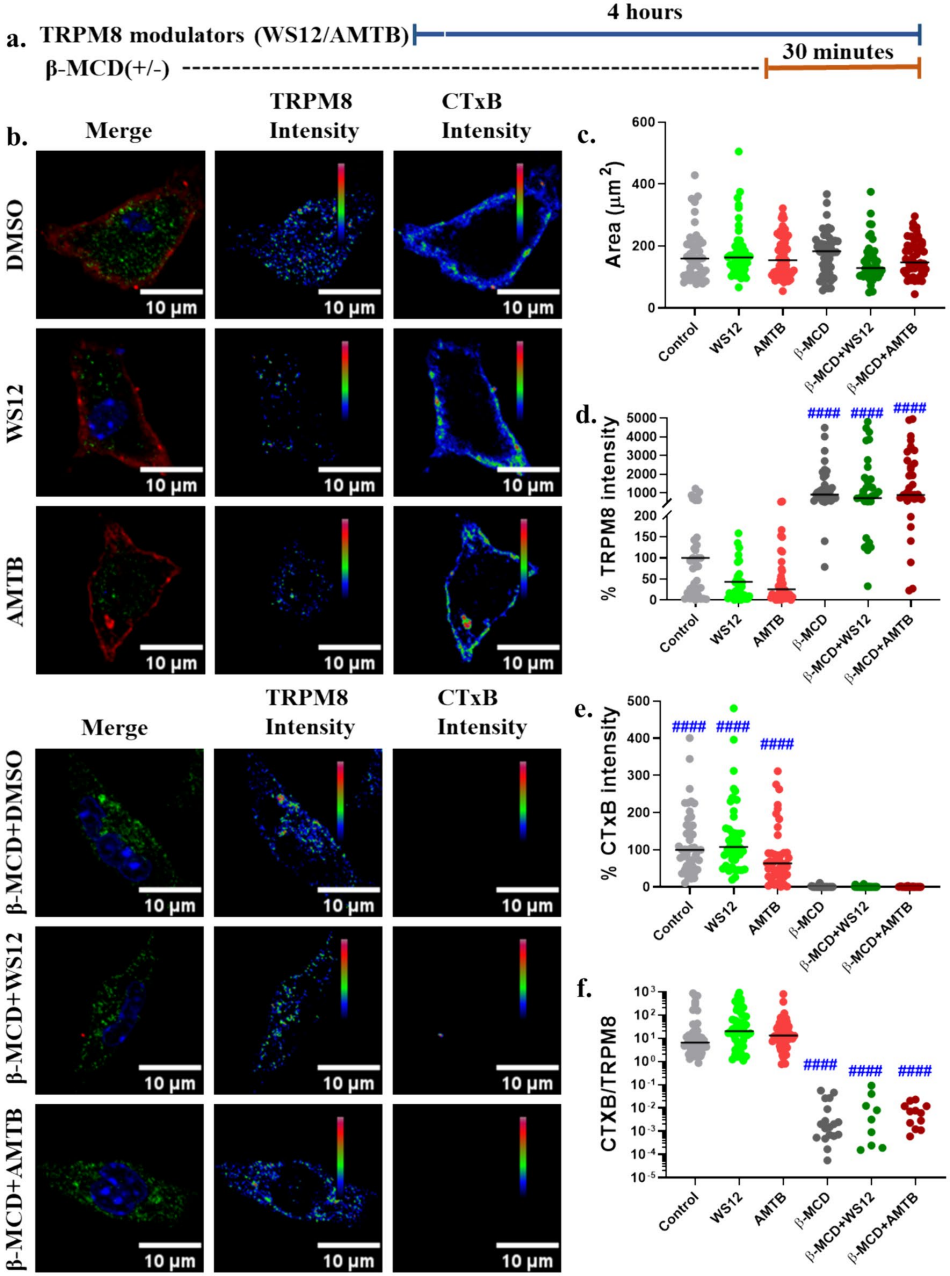
Cholesterol reduction promotes surface localization of TRPM8 in primary peritoneal macrophage. **a.** Schematic representation of the experiment and duration of treatments are depicted. **b.** Representative images show the TRPM8 intensity and cholesterol-enriched lipid raft intensity (stained with Cholera Toxin-B or CTxB). Fluorescence intensities are represented in rainbow RGB scale. The merge images represent TRPM8 in green and CTxB in red. **c.** Graph represents the cellular area after the treatment with TRPM8 modulators, with or without β-MCD treatment. **d-e.** Graphs represents % surface expression of TRPM8 (d) and Cholera Toxin-B (e). **f.** Graphical representation of ratio of CTxB and TRPM8. To calculate the statistical significance, non-parametric Kruskal–Wallis test was performed where #### = *p* < 0.0001

To study the TRPM8 correlation with cholesterol in vitro, we estimated the surface expression of TRPM8 in control and/or cholesterol reduced (by application of β-MCD) conditions. For this purpose, primary murine peritoneal macrophage has been chosen where TRPM8 expresses endogenously (Khalil et al. 2016). Cells were incubated with the TRPM8 modulators alone for 3:30 h, and subsequently cholesterol was reduced with the help of cholesterol-reducing agent β-MCD for 30 min wherever applicable (Fig. 6a). Subsequently the cells were fixed by PFA and labeling of TRPM8 at the surface was performed. At the same condition, surface expressed GM1 ganglioside was labeled with CTxB. We have observed major changes in TRPM8 surface expression due to β-MCD-treatment (Fig. 6b). In all these conditions, the cellular area remained mostly unchanged (Fig. 6c); however, the TRPM8 surface localization was significantly higher in cholesterol-reduced (β-MCD-treated) conditions, mostly irrespective of TRPM8 modulation (Fig. 6d). The surface staining for lipid-raft has also been significantly reduced in β-MCD treated condition (Fig. 6e) and so was the ratio of CTxB/TRPM8 (Fig. 6f). These data are suggestive of the major role of cholesterol in TRPM8 regulation.

### Several Mutations Causing *Cancer* are Located in the LWI and Cholesterol-Binding Regions of TRPM8

**Fig. 7:**
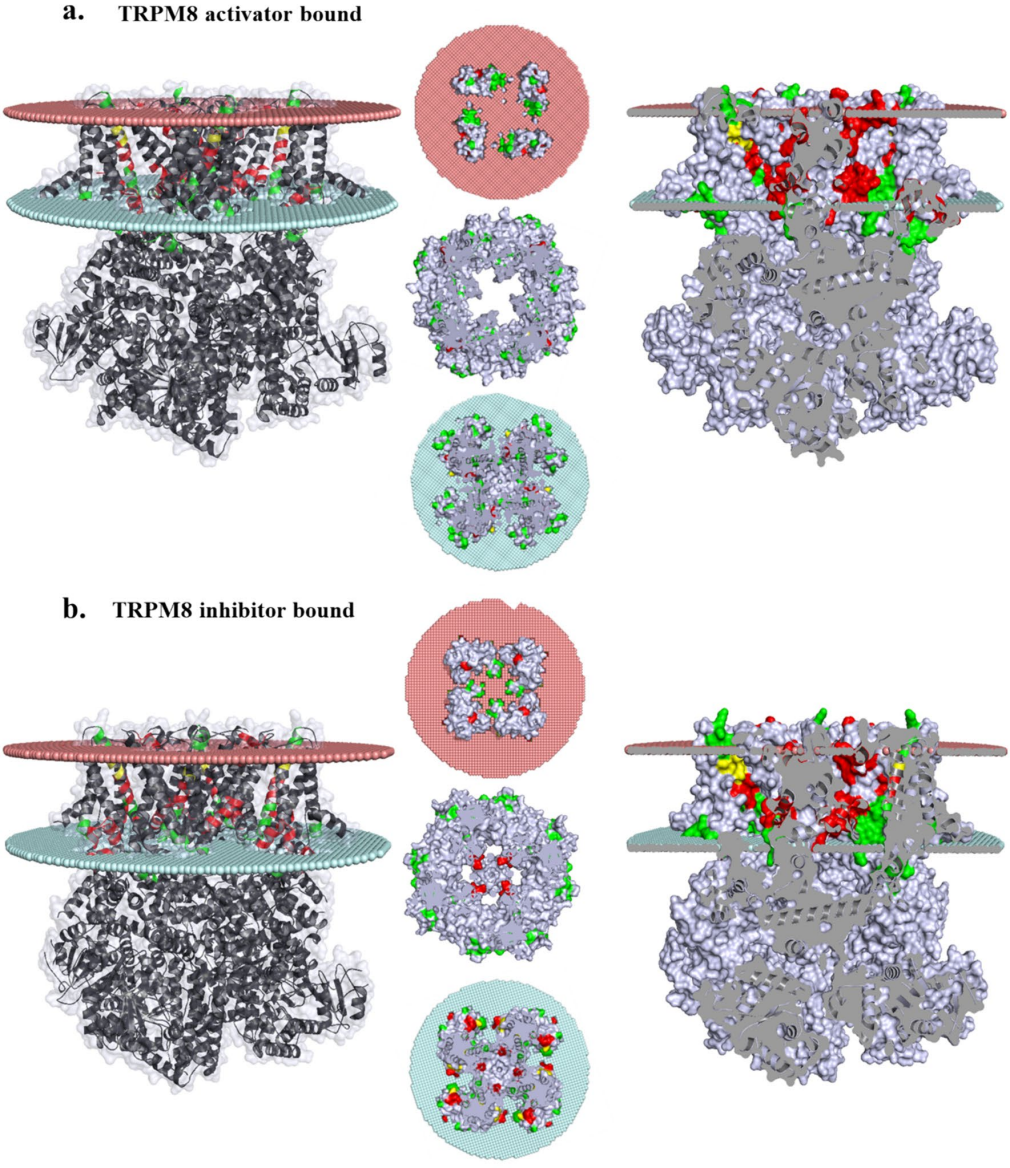
Series of somatic mutations in LWI and CARC-CRAC region of TRPM8 is found in cancer patients. The cancer mutation of LWI, and CARC-CRAC motifs are marked only in the TM regions and in LWI regions. **a-b.** A side view (left side) and transverse cut of side view (right side) of TRPM8 opened (a) and closed (b) structures with point mutations as found in cancer patients are shown. Mutations in LWI and CARC-CRAC regions only are shown in red and green respectively. The images in the middle show the top view of the structure at outer LWI (salmon color circle), the middle section of lipid bilayer and cytoplasmic region (cyan color circle). Highlighted amino acids represents the cancer mutation in LWI region (in red) and in the CARC-CRAC regions overlapping with LWI region (in yellow). These following positions, i.e. E782, P958 (for left panel, top), V784 (for right panel, top), P716, T732, S733, P734, L757, M758, V783, E832 (for left panel, below) and P734, I957 (for right panel, below) are marked in red color and these positions are located in CARC-CARC motifs where mutations in cancers are reported. Notably, V791, S827 (for left panel, top), Q785 (for right panel, top), R688, Q785, V791 (for left panel, below) and S827 (for right panel, below) are residues cancer mutations are reported and these residues indicate common positions for LWI as well as CRAC-CRAC motifs

Intracellular Ca^2+^-signaling is intimately related to the signaling of cancer cells and/or tumor progression (Cui et al. 2017). We observed a large number of mutations that are present in the TRPM8 in tumor samples as somatic mutations. We have cataloged all these somatic mutations (Supplementary Table 3). A large number of mutations have been detected in the lipid-water-interface region of TRPM8. These mutations are also observed to be present in the LWI region, both in activator as well as inhibitor-bound forms (Fig. 7a, b). Similarly, a large number of mutations are also observed in the cholesterol-binding regions. In this regard, it is known that TRPM8 is involved in testosterone signaling relevant to prostate cancer (Grolez et al. 2019). Taken together, the data strongly suggest that TRPM8 cholesterol crosstalk is important for cellular functions and alteration of such crosstalk can be relevant in different cancer conditions.

## Discussion

### The Importance of “TRPM8-Mediated Sensory Functions” is Critical for Vertebrate Evolution

In mammals, TRPM8 acts as a true sensor for low temperatures (Blanquart et al. 2019). TRPM8 also has a role in core body temperature regulation (Zhu et al. 2012). However, if TRPM8 acts as a cold-sensitive ion channel in other species, especially in the lower vertebrates, that remains an open question. Nevertheless, TRPM8 function can be correlated with the detection of cold temperature, and thus, both body temperature and environmental temperature can play an important role in the molecular evolution and/or selection of TRPM8. Our analysis of LWI regions and amino acids suggests that there are specific changes in the trend in frequency distribution of only a few amino acids (such as Val, Ile, and Leu), which can be correlated with the change in body temperature (cold- vs. warm-blooded).

### Importance of TRPM8 in Vertebrate Evolution and Molecular Evolution of TRPM8

Though at least one copy of the TRPM8 gene is present in all mammals, birds, reptilians, and amphibians, the presence of TRPM8 in fishes remains an open question, and so far TRPM8 has not been detected in the majority of fishes (where genome sequencing has been completed) with few exceptions (Kastenhuber et al. 2012; Saito and Tominaga 2015; York and Zakon 2022). On the other hand, amphibians have fully developed TRPM8 gene. Therefore, TRPM8 seems to appear during the vertebrate evolution around ca 400 million years or before.

Previously, we have demonstrated the presence of a large number of genomic variants for TRPVs as well as for TRPM8 in the human population (Ghosh et al. 2016; Kumar et al. 2015). At the protein level, there are several missense variations also, with more deleterious mutants predicted at the N-terminus. Another study also confirmed that at least 28 different splice variants of TRPM8 are present in the human population (Blanquart et al. 2019). Yet, so far, there is no deleterious mutation in TRPM8 is known that can cause “channelopathy”, and at the same time, all these variants are transmissible to the next generation, suggesting that probably all these variations are tolerable in nature.

Along with the above-mentioned variants, in this work, we have cataloged a large number of somatic mutations of TRPM8 that were found in cancer patients. Many of these mutations are typically located at the LWI regions and/or in predicted cholesterol-binding regions of the TRPM8 structure. However, the nature of these mutants, such as if these are "gain-of-function", "loss-of-function", " constitutively on" or "constitutively off", that remains an open question. Nevertheless, the fact that TRPM8 can interact with cholesterol in different modes suggests the altogether diverse and pleiotropic nature of TRPM8 and cholesterol relationships. As cholesterol is also a vertebrate-specific molecule and evolutionary appeared only in early vertebrates, our data indicates that TRPM8 has coevolved with cholesterol. In that context, cholesterol-enriched membrane seem to play important roles in the function/s of TRPM8 and its molecular selection. The high level of conservation of cholesterol-binding patches and residues within the sequence suggests that TRPM8-cholesterol may act as a functional complex. This accords well with the previous finding that suggests the reduction of membrane cholesterol redistributes the surface availability of TRPM8 (Veliz et al. 2010). In this context, it is important to note that TRPM8 functions can be regulated by testosterone (Asuthkar et al. 2015a, Asuthkar et al. 2015b).

### Amino Acids at the LWI and Cholesterol-Binding Regions

One important finding of this work is the analysis of amino acids that are present in the lipid-water- interface of TRPM8. We show that the LWI amino acids are fairly conserved, both in the outer and inner LWI regions. Though these LWI residues are derived by Protter (taking human as reference), these residues are mostly conserved in all species in terms of their location (but may have certain minor variations in different species). These minor variations can be due to the change in overall length of the TRPM8 in different species. Notably, these Protter-derived sequences also match well with the human Cryo-EM structure of TRPM8 (Figure S2). We also noted certain positional changes in LWI residues when open and close conformations were superimposed (Figure S5).

Also the ratio of hydrophilic: hydrophobic and positive: negative residues are conserved in inner as well as outer LWI regions. This aspect is a bit different than TRPV1 as well as TRPV4 (Saha et al. 2022; Das et al. 2022, 2023). Considering that TRPV1 as well as TRPV4 are older in evolutionary origin, the more conservation (in outer as well as in inner LWI region) observed in TRPM8 is striking. The ratio values of TRPM8 in the inner LWI region are hydrophilic: hydrophobic: 0.4, positive: negative: 2. Notably, this ratio values of TRPM8 differs in case of inner LWI regions of TRPV1 (hydrophilic: hydrophobic: 2.72, positive: negative: 1.68) and TRPV4 (hydrophilic: hydrophobic: 2.05, positive: negative: 6.96). However, if and how these values are related to thermo-gating, is not clear yet. Moreover, the in vitro data also suggest a potential role of cholesterol in TRPM8 function. Nevertheless, the conserved feature of TRPM8 residues in the LWI region is undoubtedly indicative of the selection pressure required for its functions. The data suggest more stringent selection pressure in the inner LWI region than that of the outer LWI region.

### Amino Acids at the LWI Region and Possible Post-Translational Modifications

The frequency analysis of all these amino acids indicates important issues of possible regulation of TRPM8 by post-translational modifications. We analyzed the Asparagine and Arginine residues present on the outer LWI region for possible N-linked glycosylation. We noted that the outer LWI region has conserved frequency of Asparagine (though lower than the natural frequency) and Arginine (much higher than the natural frequency). Similarly, we also analyzed the presence of Serine, Threonine, and Tyrosine at the outer LWI region for possible O-linked glycosylation. We noted that the frequency of Threonine and Tyrosine remain conserved (though their frequencies are just maintained at the natural frequencies). However, the frequency of Ser remains variable and no specific pattern is observed. In other words, conserved frequencies of Asparagine, Arginine, Threonine and Tyrosine suggests well possibilities of glycosylation of TRPM8during vertebrate evolution.

The post-translational modification of Cysteine residues is important for cell signalling and protein stabilization. Also, the Cysteine dimerization is very important for protein secondary structure stabilization (Zhong et al. 2023). Cysteine modification of ion channels, especially at the loop or TM regions restricts the channel's conformational flexibility leading to irreversible changes in the channel properties. Cys modification by ROS is a common for many membrane proteins (Ahmad et al. 2017). We observed that there are no Cysteine residues present at the LWI region, both at the inner as well as outer LWI region. In this regard, the complete exclusion of Cysteine from all the LWI regions of TRPM8 during the vertebrate evolution (~ 400 MY) strongly indicates that the possible occurrence of Cysteine residues in the LWI region will probably face Cysteine modification that might provide detrimental regulation or functions to TRPM8.

We also observed that in the inner LWI region of TRPM8, the frequency of Tyrosine is high, remain conserved and retained well above the natural abundance. Accordingly, the two tyrosine residues (one at N-TM5 and other at C-TM6) LWI regions remain highly conserved on the same position throughout the vertebrate evolution. This may suggest for a Tyrosine kinase mediated regulation of TRPM8 (Manolache et al. 2020).The Serine residue shows positive selection at the inner LWI region, remains at much higher frequencies than the natural frequencies and remain conserved in reptilian to mammals. The frequencies of Threonine also remain at natural abundance or more (in case of amphibians). As both Serine and Threonine act as important sites for phosphorylation by Serine-Threonine kinases (such as PKCs), the data strongly suggest that Serine and Threonine residues present in the inner LWI region of TRPM8 could be involved in regulation of TRPM8 by Serine-Threonine kinases.

### TRPM8 as a Potential Drug Target Relevant in *Cancer* Conditions, Neurodegeneration

With increasing studies, the diverse role of TRPM8 has emerged in the recent past, which suggests that TRPM8 is not merely involved in sensory functions, but also is relevant for different complex diseases. Previously we have shown the importance of TRPM8 in the T cell activation (which needs more intracellular Ca^2+^) (Acharya et al. 2021a, b). Localization of TRPM8 in lipid raft or non-raft regions of the membrane seems to be an important aspect for several disease-related conditions. As cholesterol is critically relevant for different diseases, such as in cancer conditions, immune-related conditions, and different forms of neurodegeneration. Also, the cross-talk of TRPM8-cholesterol seems to be extremely relevant for these disease conditions (Khajavi et al. 2017; King et al. 2022). It has become more relevant in disease aspects as there are endogenous factors, such as 3-iodothyronamine (T_1_AM) which can activate TRPM8 as a ligand (Pfrieger 2021). TRPM8 ion channel has been found to be crucial molecular component in progression of neurodegenerative diseases like Alzheimer's (Shaheen et al. 2021). TRPM8 is also considered a risk factor in the case of Parkinson's disease. Notably, the patients of Parkinson's disease experience a chronic pain which is found to be associated with TRPM8 gene (Williams et al. 2020). Additionally, the expression of TRPM8 gene has been reported to be upregulated in the presence of MPP (Methyl-4-phenylpyridinium), a metabolite used to induce Parkinson's disease in neuronal cell lines (Öz and Çelik 2022).

**Fig. 8:**
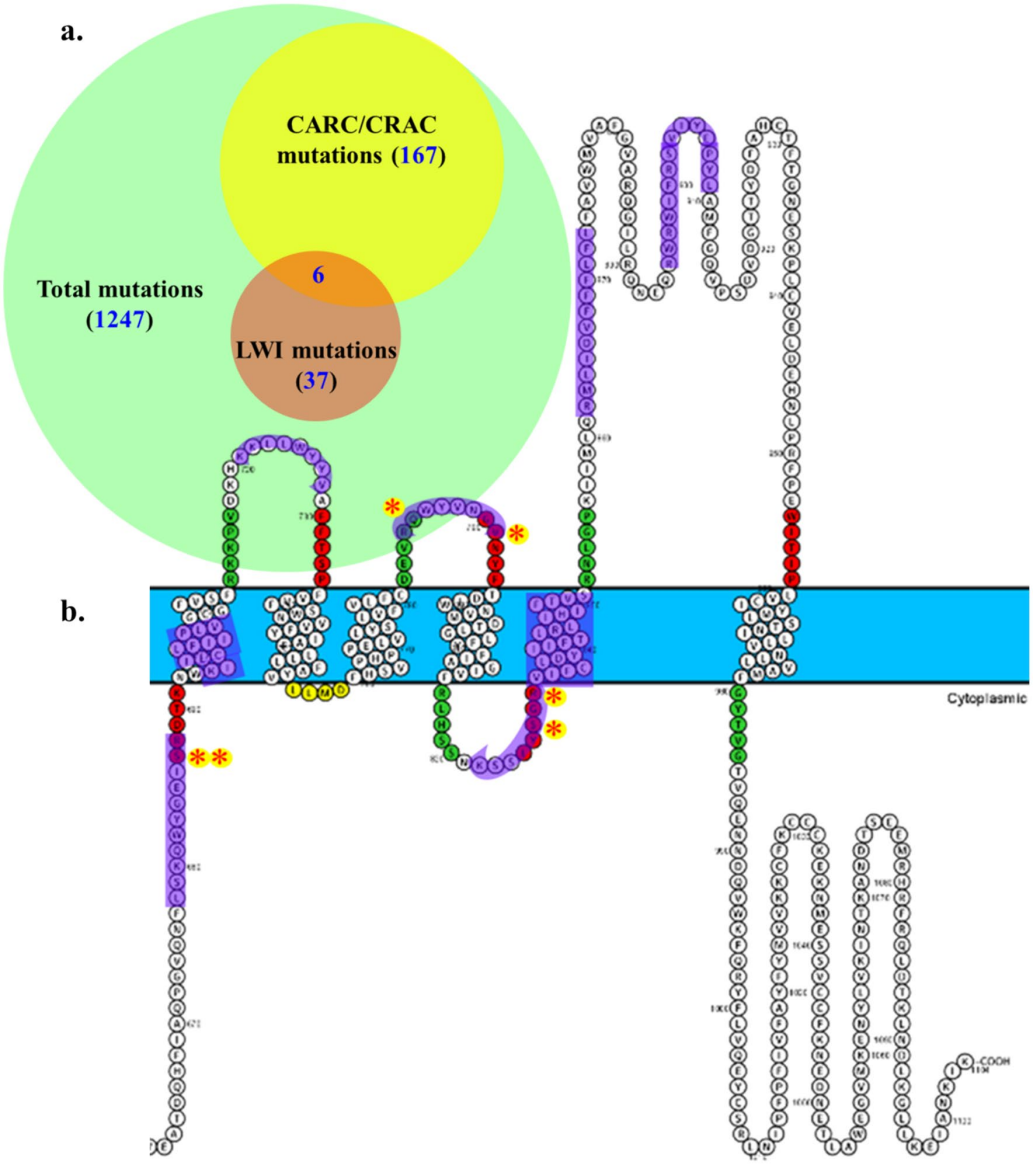
Schematic representation of LWI, CARC/CRAC regions, and somatic mutations (associated with different cancer conditions) present in TRPM8. **a.** The “Venn-diagram” shows the overall somatic cancer mutations found in TRPM8 (green circle), mutations present in CARC/CRAC regions (yellow circle), and mutations present in the LWI regions (orange circle). There are 6 mutations (namely, p.R688*, p.R688Q, p.Q785E, p.V791 = , p.S827Y, and p.R829*. (*) specifies nonsense mutation while ( =) is coding silent) which are found to be common and are positioned in CARC/CRAC and LWI. These data is derived from information available online web page COSMIC (https://cancer.sanger.ac.uk/cosmic, February, 2022). **b.** The protter image represents the LWI regions of TRPM8 (represented by red and green for N-terminal and C-terminal respectively). The CARC/CRAC regions present in the transmembrane regions are highlighted with light purple color. The mutations that overlap in both CARC/CRAC and LWI-region are highlighted with red asterisk

So far several reports have suggested that changes in the expression of TRPM8 can be linked with the development as well as progression of cancer stages. For example, TRPM8-mediated proliferation has been observed in esophageal cancer cell lines (Lan et al. 2019). Migration and invasion of squamous carcinoma cell is more in case of TRPM8 activation and less in case of inhibition (Okamoto et al. 2012). In case of bladder cancer, TRPM8 expression is increased in cancerous tissue (Wang et al. 2020). TRPM8 expression level in pancreatic cancer tissue has been found to be moderate to high (related to the stage of pancreatic cancer), and depends on the distance of metastasis and tumor size. Also higher expression TRPM8 has been proposed for reduced survival (Du et al. 2018). Consistent with these findings, the data obtained by COSMIC suggest various cancer mutations (1247) throughout TRPM8 structure. Out of which, 161 found in the CARC/CRAC regions, and 37 was spotted in the LWI regions. Among these mutation, 6 mutations are found in an overlapping regions of the CARC/CRAC and LWI (Fig. 8a, b). In this context, it is important to note in general cholesterol level is high in cancer conditions, and statin drugs are often used along with the chemotherapeutics (Kuzu et al. 2016). In addition to that, it is also observed that the lipid symmetry and cholesterol content of the membrane changes in cancerous conditions (Rivel et al. 2019). It is also important to note that cancer patients seem to have diverse somatic mutations in TRPM8.

### Limitations of this Study

The primary findings and all the interpretation mentioned in this manuscript is based on the sequence analysis of 67 different vertebrate species that was available till the year 2022 (Supplementary Table 1). Due to the rapid update in the genome sequence, the interpretation of our findings may vary in the future. Also actual calculation of all these somatic mutations at the codon level may provide useful information about the mutational frequencies acting on the LWI region as compared to other regions. For the updated results, there is a need for periodic updates in the analysis of the latest available sequences. Also, the behavior of mutants that are common for the CARC/CRAC region as well as for the LWI region needs to be verified experimentally in the future. Further work with point mutants can also validate the exact role of individual amino acids with respect to cholesterol interaction and regulation.

## Conclusion and Future Aspects

Collectively, our data suggests that the positive-to-negative ratio, and hydrophobic-to-hydrophilic ratio of the LWI regions have a pattern and such pattern (especially the pattern of the inner LWI) may be important for channel function. TRPM8 seems to co-evolved with cholesterol-binding motifs, which is suggestive of a complex relationship of TRPM8 with cholesterol. Accordingly, at least as a proof-of-principle, we show that membrane cholesterol level regulates the surface expression of TRPM8 in primary macrophages. Therefore, TRPM8- cholesterol– crosstalk may have broad implications in several diseases and in clinical treatment conditions associated with TRPM8.

## Supplementary Information

Below is the link to the electronic supplementary material.

Supplementary file1 (DOCX 3638 KB)

## Data Availability

No datasets were generated or analyzed during the current study. All data generated or analyzed in this study are included in this manuscript [and its supplementary information files]. The additional datasets analyzed in the current study can be available from the corresponding author on reasonable request.
