# Supplementary material for: Residues of TRPM8 at the Lipid-Water-Interface have Coevolved with Cholesterol Interaction and are Relevant for Diverse Health Disorders
Source: J Membr Biol. 2024 Aug 16;257(5-6):345–64. doi: 10.1007/s00232-024-00319-y (PMC11584472; doi:10.1007/s00232-024-00319-y)
Supplement: Supplementary file 1 — Supplementary file1 (DOCX 3638 KB) [file 232_2024_319_MOESM1_ESM.docx]

**Supplementary figures, tables and legends**

**Residues of TRPM8 at the lipid-water-interface have coevolved with cholesterol interaction and are relevant for diverse health disorders**

**Deep Shikha, Ritesh Dalai, Shamit Kumar, Chandan Goswami**

School of Biological Sciences, National Institute of Science Education and Research, An OCC of Homi Bhabha National Institute, Khordha, Jatni, Odisha, 752050, India

Correspondence: [chandan@niser.ac.in](mailto:chandan@niser.ac.in)

**Supplementary figures and legends**

**
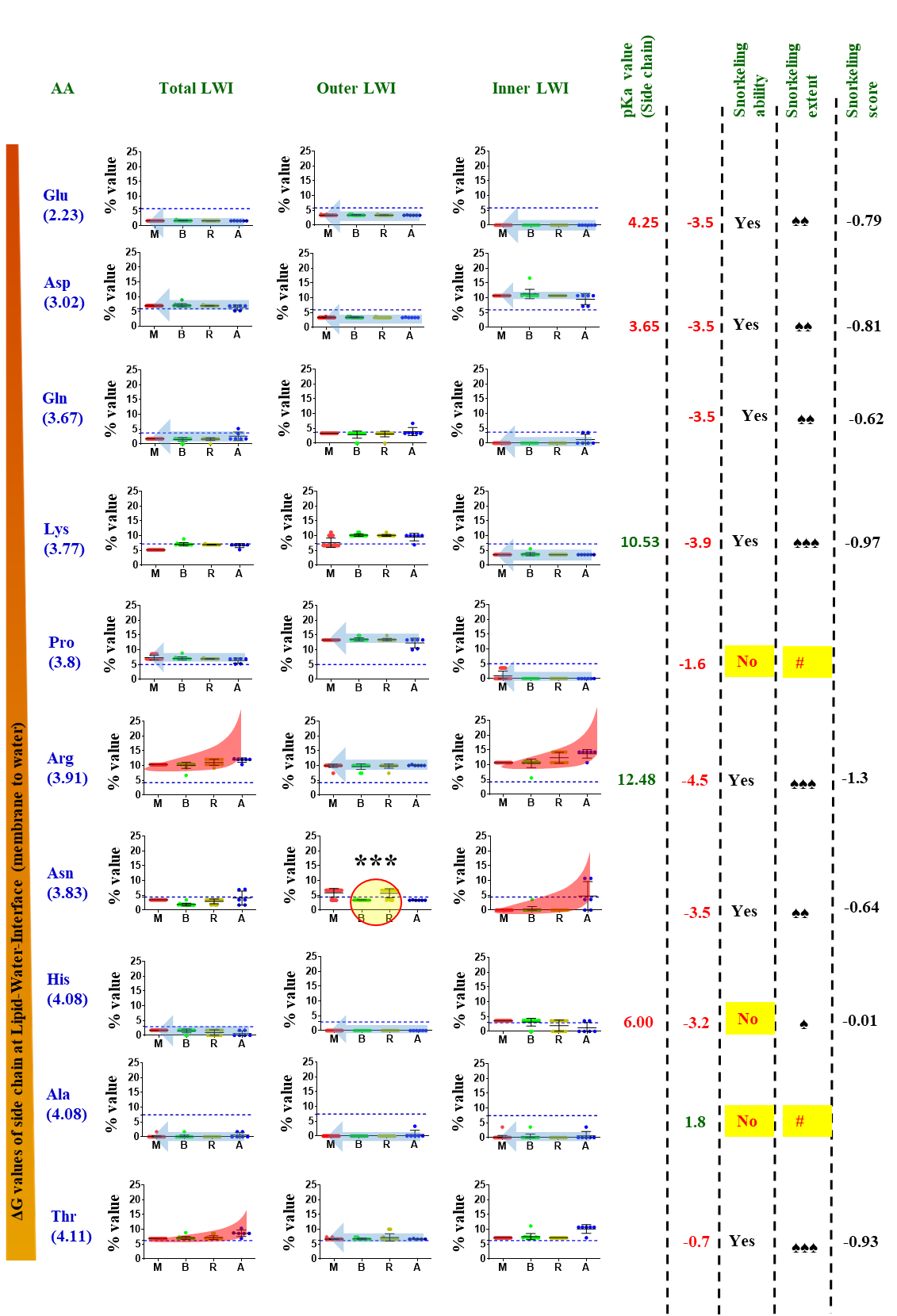
**

**
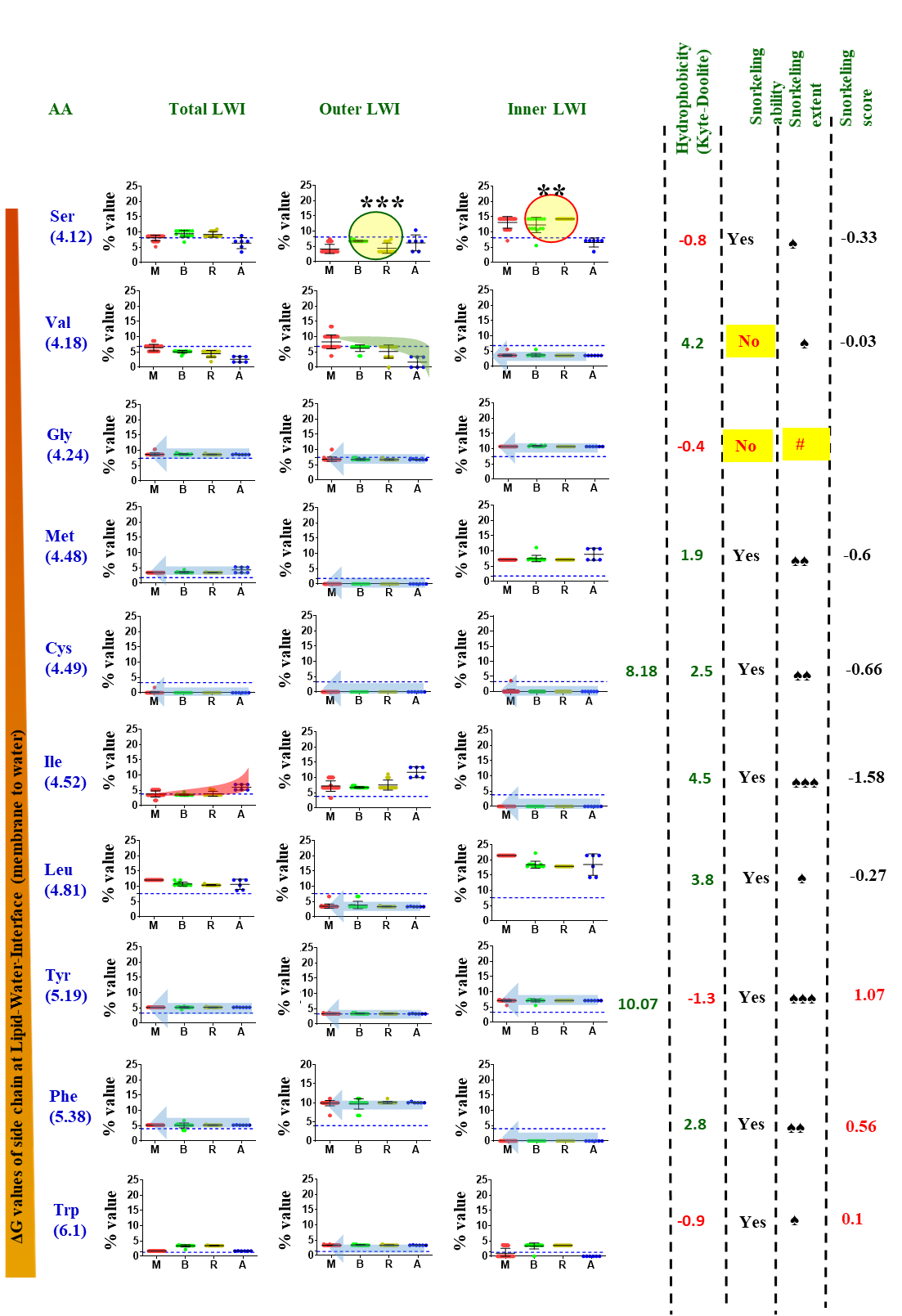
**

**Fig S1: Frequency analysis of individual amino acids in LWI region of TRPM8 in different species.** A representation of frequency of individual amino acid in total LWI, outer LWI and inner LWI regions. Blue dotted line represents the natural frequency of individual amino acids. A positive selection of an amino acid in the bird over reptile has been encircled in green while the negative selection is highlighted in red circle. The overall positive selection from amphibians towards mammals has been highlighted with green and the reverse in red. Statistical significance has been calculated by Mann-Whitney t-test between mammals-birds and birds-reptiles. Where, ****= p< 0.0001 and ***= p< 0.001.

| **Regions** | **Human-Protter** | **Human-PDB (8BDC)** | **Mismatch%** |
| --- | --- | --- | --- |
| TM1N | SRDTK | SRDTK | 0 |
| TM1 | NWKIILCLFIIPLVGCGFVSF | NWKIILCLFIIPLVGCGFVSF | 0 |
| TM1C | RKKPV | RK**x**PV | 20 |
| TM2N | FFTSP | FFTSP | 0 |
| TM2 | FVVFSWNVVFYIAFLLLFAYV | FVVFSWNVVFYIAFLLLFAYV | 0 |
| TM2C | LLMD | LLMD | 0 |
| TM3N | LLMD | LLMD | 0 |
| TM3 | FHSVPHPPELVLYSLVFVLFC | FHSVPHPPELVLYSLVFVLFC | 0 |
| TM3C | DEVRQ | **x**EVRQ | 20 |
| TM4N | GVNYF | G**xx**YF | 40 |
| TM4 | TDLWNVMDTLGLFYFIAGIVF | TDLWNVMDTLGLFYFIAGIVF | 0 |
| TM4C | RLHSS | RLHS**x** | 20 |
| TM5N | LYSGR | LYSGR | 0 |
| TM5 | VIFCLDYIIFTLRLIHIFTVS | VIFCLDYIIFTLRLIHIFTVS | 0 |
| TM5C | RNLGP | RNLGP | 0 |
| TM6N | WITIP | WITIP | 0 |
| TM6 | LVCIYMLSTNILLVNLLVAMF | LVCIYMLSTNILLVNLLVAMF | 0 |
| TM6C | GYTVG | GYTVG | 0 |

NOTE: The x represents the Residues that don't have coordinates of all of their atoms.


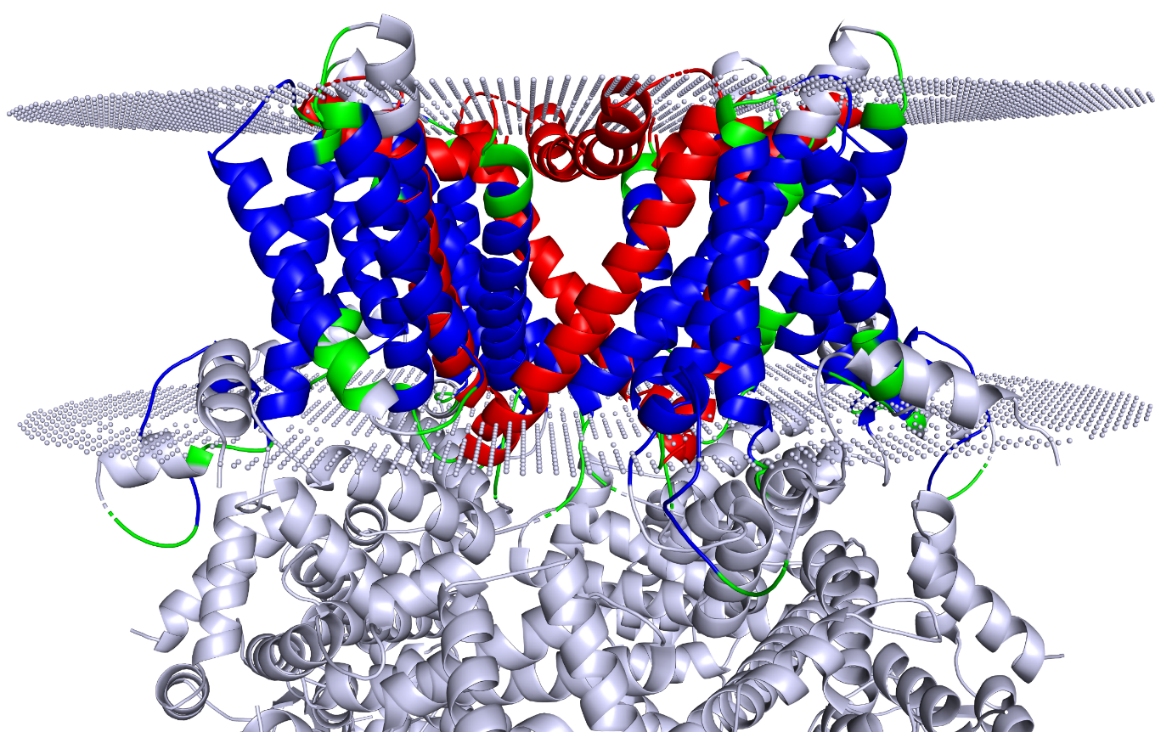


**Fig S2: Comparision of LWI residues in human TRPM8.** The above table indicates the residues as identified in Protter and in PDB strcuture. In below, the LWI residues (green), TM regions (blue) and loop regions (red) of the human TRPM8 are indicateds.

**
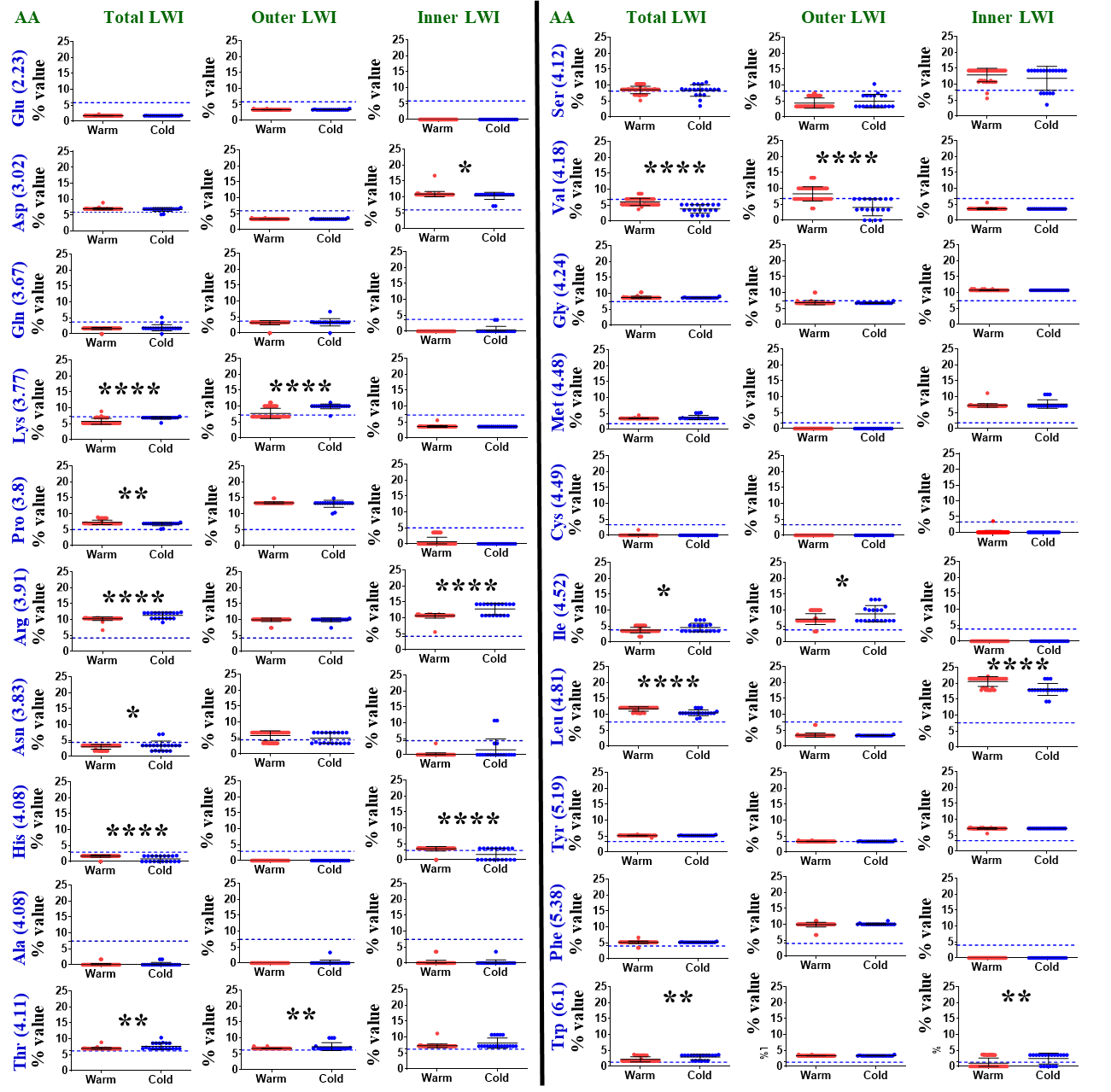
**

**Fig S3: Percentage conservation of individual amino acids in LWI region in warm and cold blooded animals.** The individual amino acid frequency in total, outer and inner LWI. The warm blooded animals are represented in red and cold blooded animals are represented in blue. The blue dotted line represents the natural frequency of individual amino acid. Mann-Whitney test was performed to calculate statistical significance, where, * = p<0.01, ** = p<0.001, **** = p<0.0001. Total number of sequences used are 48 for warm blooded animals and 19 for cold-blooded animals. In spite of p values, only in selected few cases (such as Val, Ile, Leu), the difference in the mean value between warm and cold-blooded animals are observed.

**
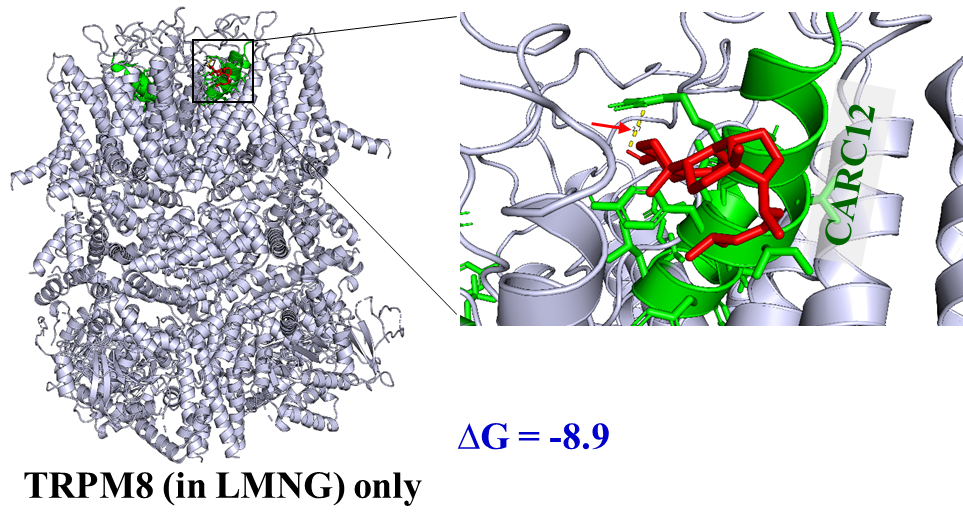
**

**Fig S4: Cholesterol interaction with ligand-free mouse TRPM8 (Cryo-EM structure, PDB ID: 7WRA)**. Cholesterol (red) docking with mouse TRPM8 Cryo-EM structure in ligand-free state shows docking at CARC12 (green).

**
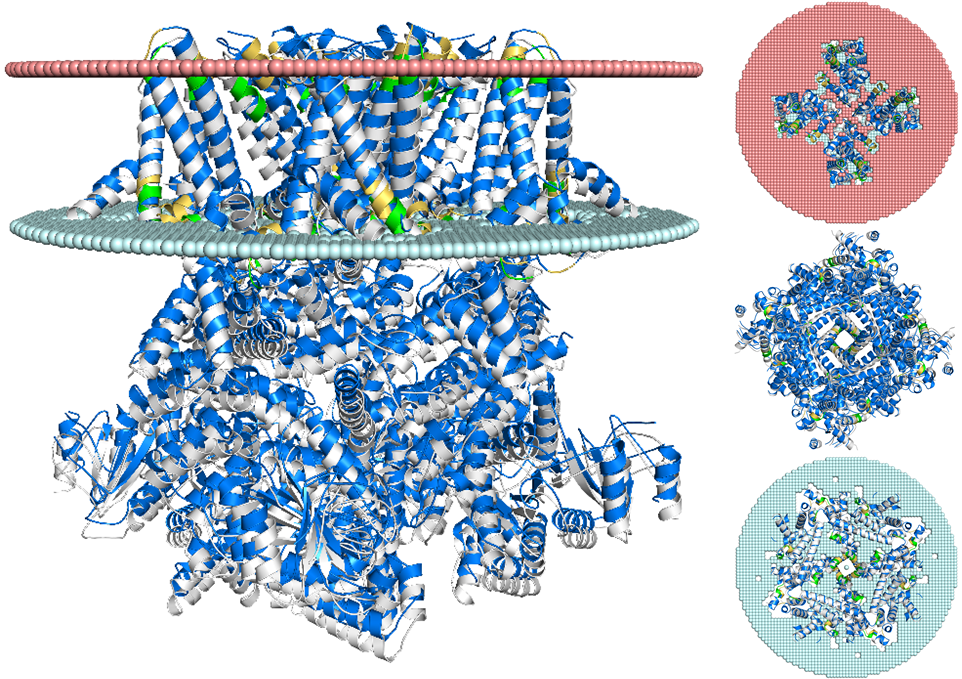
**

**Fig S5: Change in LWI positions in open, and close structure of TRPM8.** The image shows the merged images of the TRPM8 in open (white) and close (blue) conformation. Here, LWI regions of open conformation are shown in green, and LWI regions of close conformation are shown in gold.

**Supplementary Table 1.**

**Birds**

**Reptiles**

| **Species Name** | **Accession no.** | **Sequence length (amino acid)** | **Species Name** | **Accession no.** | **Sequence length (amino acid)** |
| --- | --- | --- | --- | --- | --- |
| *Homo sapiens* | NP_076985.4 | 1104 | *Hyaena hyaena* | XP_039090393.1 | 1104 |
| *Rattus norvegicus* | NP_599198.2 | 1104 | *Lynx canadensis* | XP_032450057.1 | 1104 |
| *Mus musculus* | NP_599013.1 | 1104 | *Sturnira hondurensis* | XP_036925089.1 | 1104 |
| *Saimiri boliviensis boliviensis* | XP_010345521.2 | 1104 | *Pteropus giganteus* | XP_039715880.1 | 1104 |
| *Chlorocebus sabaeus* | XP_007964912.1 | 1104 | *Artibeus jamaicensis* | XP_037017351.1 | 1104 |
| *Cebus imitator* | XP_017393202.1 | 1104 | *Molossus molossus* | XP_036109259.1 | 1104 |
| *Callithrix jacchus* | XP_002749959.1 | 1104 | *Rousettus aegyptiacus* | XP_016014442.2 | 1104 |
| *Papio anubis* | NP_001162392.1 | 1104 | *Rhinolophus ferrumequinum* | XP_032967298.1 | 1104 |
| *Pan paniscus*  **Mammals** | XP_034811295.1 | 1104 | *Aquila chrysaetos chrysaetos* | XP_029874684 | 1104 |
| *Hylobates moloch* | XP_032609565.1 | 1104 | *Falco naumanni* | XP_040460744 | 1104 |
| *Sapajus apella* | XP_032133738.1 | 1104 | *Cygnus olor* | XP_040416904 | 1103 |
| *Piliocolobus tephrosceles* | XP_023084498.1 | 1104 | *Anas platyrhynchos* | XP_038038349 | 1107 |
| *Gorilla gorilla gorilla* | XP_030864771.1 | 1104 | *Egretta garzetta** | XP_009633769 | 1104 |
| *Nomascus leucogenys* | XP_003278601.1 | 1104 | *Parus major* | XP_033371797 | 1103 |
| *Theropithecus gelada* | XP_025260385.1 | 1104 | *Coturnix japonica* | XP_015723255 | 1104 |
| *Macaca nemestrina* | XP_011726325.1 | 1104 | *Meleagris gallopav* | XP_019472396 | 1104 |
| *Pongo abelii* | XP_009236533.1 | 1104 | *Athene cunicularia* | XP_026708792 | 1104 |
| *Mesocricetus auratus* | AUF73696.1 | 1104 | *Columba livia* | XP_005513174.1 | 1104 |
| *Ictidomys tridecemlineatus* | AUF73695.1 | 1104 | *Taeniopygia guttata* | XP_041573467.1 | 1104 |
| *Cavia porcellus* | NP_001166561.1 | 1104 | *Hirundo rustica* | XP_039925973.1 | 1103 |
| *Dipodomys spectabilis* | XP_042535087.1 | 1104 | *Motacilla alba alba* | XP_037999400.1 | 1103 |
| *Peromyscus maniculatus bairdii* | XP_006988706.3 | 1104 | *Molothrus ater* | XP_036243104.1 | 1113 |
| *Microtus oregoni* | XP_041506419.1 | 1104 | *Mauremys reevesii* | XP_039350632.1 | 1098 |
| *Canis lupus familiaris* | ABB89753.1 | 1104 | *Dermochelys coriacea* | XP_038277665.1 | 1097 |
| *Vulpes lagopus* | XP_041623532.1 | 1104 | *Chelonia mydas* | XP_037768935.1 | 1095 |
| *Puma yagouaroundi* | XP_040306547.1 | 1104 | *Trachemys scripta elegans* | XP_034641644.1 | 1098 |

| **Species Name** | **Accession no.** | **Sequence length (amino acid)** | **Species Name** | **Accession no.** | **Sequence length (amino acid)** |
| --- | --- | --- | --- | --- | --- |
| *Chelonoidis abingdonii* | XP_032627399.1 | 1098 | *Lacerta agilis* | XP_033028319.1 | 1095 |
| *Gopherus evgoodei* | XP_030436697.1 | 1098 | *Xenopus laevis* | XP_018089453.1 | 1139 |
| *Pelodiscus sinensis* | XP_014431134.1 | 1055 | *Bufo bufo* | XP_040296203.1 | 1139  **Amphibians** |
| *Chrysemys picta bellii* | XP_008170810.2 | 1041 | *Xenopus tropicalis* | NP_001155105.1 | 1141 |
| *Sceloporus undulatus* | XP_042303008.1 | 1092 | *Geotrypetes seraphini* | XP_033799988.1 | 1108 |
| *Crotalus tigris* | XP_039204983.1 | 1092 | *Microcaecilia unicolor* | XP_030066681.1 | 1071 |
| *Zootoca vivipara* | XP_034995026.1 | 1104 | *Rana temporaria* | XP_040213489.1 | 1141 |
| *Pantherophis guttatus* | XP_034282114.1 | 1044 |  |  |  |

**Reptiles**

Accession Number and amino acid sequence length of TRPM8 proteins Accession number of mammals, birds, reptiles and amphibians are given in green, blue, yellow and orange boxes respectively.

**Supplementary Table 2.**

| **AA range** | **CRAC- Sequence** | **AA range** | **CARC-Sequence** | **AA range** | **CARC-Sequence** |
| --- | --- | --- | --- | --- | --- |
| 1058-1066 | **V**M**KE**NYLVK | 1079-1085 | RHRFRQL | 513-521 | **K**N**SY**ND**ALL** |
| 1017-1026 | **VFA**Y**F**Y**M**V**VK** | 1060-1065 | KENYLV | 485-492 | **R**KF**LT**HDV |
| 1017-1027 | **VFA**Y**F**Y**M**V**VK**K | 1008-1017 | RLNIPFPFIV | 485-493 | **R**KF**LT**HDVL |
| 1002-1008 | VQEYCS**R** | 998-1002 | RYFLV | 475-482 | **R**LFL**E**N**G**L |
| 1001-1008 | LVQEYCS**R** | 995-1001 | KFQRYFL | 472-482 | KFV**R**LFL**E**N**G**L |
| 834-842 | LD**Y**IIF**TLR** | 995-1002 | KFQRYFLV | 470-476 | R**P**KFV**R**L |
| 830-842 | VIF**C**LD**Y**IIF**TLR** | 901-909 | RSVIYEPYL | 470-478 | R**P**KFV**R**LFL |
| 678-688 | **L**SK**Q**WY**G**EIS**R** | 897-903 | RWIFRSV | 468-476 | K**D**R**P**KFV**R**L |
| 617-624 | L**A**NEYE**TR** | 895-903 | RWRWIFRSV | 468-478 | K**D**R**P**KFV**R**LFL |
| 404-414 | **V**SN**A**ISYA**LY**K | 862-871 | RMLIDVFFFL | 357-366 | K**L**V**R**FL**PRTV** |
| 281-288 | L**E**KYI**SER** | 862-873 | RMLIDVFFFLFL | 355-366 | K**E**K**L**V**R**FL**PRTV** |
| 188-197 | L**M**KYIGEVV**R** | 842-849 | RLIHIFTV | 247-253 | **R**D**PLYIL** |
| 180-190 | **LTG**G**T**HYGL**M**K | 842-853 | RLIHIFTVSRNL | 163-168 | **KI**FS**RL** |
| 168-175 | **L**IY**IA**Q**SK** | 829-834 | RVIFCL | 162-168 | **RKI**FS**RL** |
| 111-119 | LG**K**K**G**K**Y**I**R** | 822-830 | KSSLYSGRV | 160-168 | **R**M**RKI**FS**RL** |
|  |  | 784-791 | RQWYVNGV | 153-157 | **KNFA**L |
|  |  | 722-728 | KLLWYYV | 114-120 | K**G**K**Y**I**RL** |
|  |  | 721-728 | KKLLWYYV | 113-120 | **K**K**G**K**Y**I**RL** |
|  |  | 694-704 | KIILCLFIIPL | 30-41 | **R**STDLSY**S**E**SD**L |
|  |  | 694-705 | KIILCLFIIPLV | 30-42 | **R**STDLSY**S**E**SD**L**V** |
|  |  | 513-520 | KNSYNDAL |  |  |

**List of CRAC and CARC regions in TRPM8 structure with their amino acid number in the TRPM8 sequence.** List of CARC and CARC sequences are shown in red and green boxes, respectively. The CARC and CARC regions present in the LWI regions are presented with red and green texts respectively. Bold red residues show the somatic cancer mutations.

**Supplementary Table 3.**

| p.R30Q | p.L168= | p.R288C | p.R485L | p.P703= | p.R897K | p.V1025= | p.M758I |
| --- | --- | --- | --- | --- | --- | --- | --- |
| p.R30= | p.I171= | p.R288H | p.L488F | p.V705E | p.F900= | p.K1026N | p.M758I |
| p.S37N | p.A172= | p.E356K | p.T489= | p.K721R | p.R901C | p.V1058= | p.T732N |
| p.S39= | p.S174Y | p.L358P | p.K513= | p.Y726F | p.S902* | p.K1060M | p.T732I |
| p.D40N | p.K175N | p.R360H | p.K513N | p.V728A | p.S902L | p.E1061K | p.S733Y |
| p.V42A | p.L180F | p.P363S | p.S515F | p.Q785E | p.I904M | p.E1061D | p.S733= |
| p.V42= | p.T181M | p.P363= | p.Y516C | p.V791= | p.Y905= | p.R1079S | p.P734S |
| p.K113N | p.G182E | p.R364C | p.A519= | p.S824C | p.L909M | p.H1080L | p.P734L |
| p.G115E | p.T184I | p.R364H | p.L520= | p.S827Y | p.K995N | p.H1080= | p.R713K |
| p.Y117C | p.M189I | p.R364L | p.L520= | p.R829* | p.Q997R | p.R1081* | p.R713= |
| p.R119S | p.R197G | p.T365M | p.L521= | p.C833del | p.F1000= | p.L1085P | p.P716L |
| p.R119C | p.R247T | p.V366M | p.A618D | p.Y836= | p.R1008C | p.G980V | p.R688* |
| p.R119H | p.P249Q | p.V404= | p.T623= | p.T840= | p.R1008H | p.Y981D | p.R688Q |
| p.L120= | p.P249L | p.A407D | p.R624W | p.L841V | p.L1009= | p.T982= | p.T690I |
| p.K153N | p.P249= | p.A407= | p.R624Q | p.R842K | p.P1012S | p.I957V | p.K691= |
| p.N154S | p.L250= | p.L412V | p.L678V | p.M863I | p.P1012L | p.R829* | p.K691N |
| p.F155= | p.Y251C | p.Y413H | p.Q681= | p.L864P | p.I1016= | p.R851I |  |
| p.A156T | p.I252M | p.D469H | p.G684R | p.L864= | p.V1017I | p.N852S |  |
| p.A156= | p.L253M | p.P471T | p.R688* | p.I865= | p.V1017= | p.N852K |  |
| p.R160C | p.E282D | p.P471H | p.R688Q | p.F870S | p.V1017= | p.G854* |  |
| p.R160H | p.S286Y | p.R475H | p.K694* | p.L871= | p.F1018= | p.E782K |  |
| p.R162C | p.S286C | p.E479G | p.L697= | p.L871= | p.A1019T | p.V783= |  |
| p.K163N | p.S286F | p.E479V | p.L697Q | p.L873F | p.F1021L | p.Q785E |  |
| p.I164N | p.E287= | p.G481D | p.L699V | p.R895C | p.M1023L | p.V791= |  |
| p.R167Q | p.R288S | p.R485Q | p.P703H | p.R895H | p.M1023I | p.L757I |  |

**List of somatic cancer mutations in CARC-CRAC and LWI region of TRPM8 respectively.** The list of somatic cancer mutations in CRAC-CARC and LWI regions are represented in green and orange boxes respectively. Here, (*) specifies nonsense mutation while (=) is coding silent.
